# Regional differences in acute hospitalization risk associated with NO_2_ by cause, season, age, sex, and trend: an ecological time series study in Canada

**DOI:** 10.1186/s12889-025-22339-6

**Published:** 2025-03-31

**Authors:** Hwashin Hyun Shin, James G. Owen, Kimberly Megan Mitchell, Marc Smith-Doiron, Parvin Dehghani

**Affiliations:** 1 Environmental Health Science and Research Bureau, Health Canada, 269 Laurier Ave. W., Ottawa, ON K1A 0K9 Canada; 2 Department of Mathematics and Statistics, Queen’s University, Kingston, ON Canada

**Keywords:** Acute hospitalization, Air pollution, Bayesian hierarchical model, Nitrogen dioxide, Over-dispersed generalized Poisson models, Regional risk

## Abstract

**Background:**

Nitrogen dioxide (NO_2_) is a highly reactive gas produced mainly from burning fossil fuels. Exposure to NO_2_ has been shown to impact public health worldwide. However, spatial and temporal variations in its effects by season, age, and sex have been underexamined.

**Methods:**

We conducted an ecological time-series study based on about 20 million people (52% of Canadians in 2012) in three regions (Western, Central and Eastern Canada) over 17 years (1996–2012). We collected hourly NO_2_ concentrations and temperatures, and daily counts of non-accidental all-cause, circulatory-, and respiratory-related hospitalizations, including more specific causes: ischemic heart disease, other heart disease, cerebrovascular disease, influenza/pneumonia, and chronic lower respiratory disease. We first estimated city-specific risks, applying over-dispersed generalized Poisson models, and then regional and national risks for each season, age-group, and sex using Bayesian hierarchical models. We also applied Sen’s test to detect linear trends in annual regional and national risks.

**Results:**

We found significant NO_2_ effects by cause, season, age, sex, and linear trend. For circulatory hospitalization, only Western Canada showed significant adverse effects for non-seniors (≤ 65) (1.7% with 95% credible interval of 0.3–3.2% per 10 ppb increase in NO_2_), and for males for more specific cause, ischemic heart disease (2.3%, 0.1–4.5%). Regional differences were observed for circulatory but not respiratory hospitalizations. For example, the Western and Eastern regions were at significantly higher risk of circulatory hospitalization but not the Central region: 1.6% (0.2–3.0%) for the Western region; 2.0% (0.6–3.4%) for the Eastern region; and 0.8% (-0.3–2.0%) for the Central region. In particular, the Western region had a much higher risk of cerebrovascular disease hospitalization: 2.8% (1.1–4.6%) for the Western region; 0.1% (-3.0–3.1%) for the Central region; and 0.0% (-3.4–3.5%) for the Eastern region. However, no other regional differences were observed for other causes. Overall, there were noticeable increases in regional differences over time, particularly in the later years.

**Conclusions:**

This study indicates harmful NO_2_ effects on acute hospitalizations year-round: circulatory causes (cold season) and respiratory causes (warm season). Future work is warranted to investigate potential causes of observed regional differences using more community-related information such as socioeconomic status, health-care accessibility, and others.

**Supplementary Information:**

The online version contains supplementary material available at 10.1186/s12889-025-22339-6.

## Background

Nitrogen dioxide (NO_2_) is a highly reactive gas and important air pollutant known to affect human health. The combustion of fossil fuels is the main anthropogenic source of NO_2_ from both ambient (motor vehicles, fossil fuel power plants, and industrial sources) and indoor (appliances such as gas stoves, heaters, fireplaces, furnaces and generators) areas. NO_2_ is the ambient air pollutant most strongly linked to mortality in Canada, reported by Shin et al. [1] and Burnett et al. [2]. Daily NO_2_ exposure impacts short-term human health conditions worldwide and have been associated with non-accidental all-cause mortality, in Canada by Brook et al. [3] and Spain by Linares et al. [4], and hospitalization in northern Italy by Carugno et al. [5]. Associations with cause-specific health effects such as cardiovascular [6–8], respiratory [9, 10], and cerebrovascular disease [5] have also been widely studied. However, not all studies have found significant associations: In separate works, Staffoggia & Bellander and Koken et al. reported that NO_2_ was not associated with all-cause mortality in Stockholm, Sweden [11] or cardiovascular hospitalizations among individuals aged 65 years and older in Denver, USA [12].

Increasing efforts have been placed on investigating differences in adverse health effects of NO_2_ by season, cause, age, sex, and location (e.g., country). In the warm season, two multi-city studies in Canada reported stronger associations with non-accidental mortality than the cold season [2, 3], and another Canadian study observed greater risks of both cardiopulmonary (CP) and non-CP mortality [1]. Risks associated with respiratory hospitalization and mortality were stronger in the warm season in a recent study of 24 cities across Canada [13]. Internationally, there were greater effects of NO_2_ on hospitalizations (cardiac, cerebrovascular, respiratory) and mortalities (all-cause, cardiovascular) in the warm season in Italy [5, 10]. Associations with mortality in Stockholm County, though not significant, were stronger in the warm season [11]. Goldberg et al. reported that among Montreal seniors with acute coronary artery disease in the year before death, there was increased daily mortality associated with NO_2_ in the warm season [14]. Studies by Amini et al. and Chen et al. have reported slightly stronger associations with mortality in the cold season (or cool period), especially for men, in Iran [15] and China with cardiovascular and respiratory mortality [6]. A recent study found a season-sex-specific effect of NO_2_: males were at a higher risk of hospitalization for ischemic heart disease in the warm season, but females were at a higher risk for other heart diseases in the cold season [16]. However, Meng et al.’s broad international study of 398 cities found no evidence of seasonal differences in the association between NO_2_ and mortality [7].

Location (country or region) may also influence the effects of NO_2_ on hospitalization and mortality. In Southern China, associations with mortality were more than double those in the North [6]. Another China study led by Zeng et al. [17] examined further geographic features in a mountainous megacity, Chongqing, with dense populations and high humidity and reported that NO2 exposure could worsen the risk of influenza infection in the mountainous city. In Canada, from 1984 – 2004, regional differences in short-term effects were not apparent; however, an increasing linear time trend in the annual between-region heterogeneities suggests the differences may be increasing [1]. Differences in associations between regions may be explained by higher average temperatures and discrepancies in daily variations of NO_2_ concentrations, health care systems, and demographic characteristics. Another Canadian study, by Crouse et al. [9] found that most associations between NO_2_ and mortality were determined by within-city contrasts for long-term exposure effect, as opposed to between-city contrasts in NO_2_. However, they could not examine regional differences based on 10 large Canadian cities. The inconsistent findings in NO2-related hospitalizations might be related to environmental background such as temperature and geographic features (mountains and valleys, etc.), which all influence NO2 interactively and then hospital admissions collectively to a greater degree than NO2 alone.

Overall, there is compelling evidence suggesting that cause, season, age, sex, and location are important factors in understanding the effects of short-term exposure to NO_2_ on human health [16]; however, the current body of work regarding spatial variations and temporal trends in the associations between NO_2_ and hospitalization is limited. In this study, we investigate those factors in three ways: (1) examining regional differences in the adverse health effects of NO_2_ by season, age, and sex; (2) focusing on hospital admissions for three broad causes in addition to five specific causes; and (3) detecting trends with dynamic models during the 17-year study period from 1996 to 2012. In this article, we consider short-term exposures, non-accidental hospitalizations, and linear trends, unless otherwise specified.

## Methods

### Study design

**Fig. 1:**
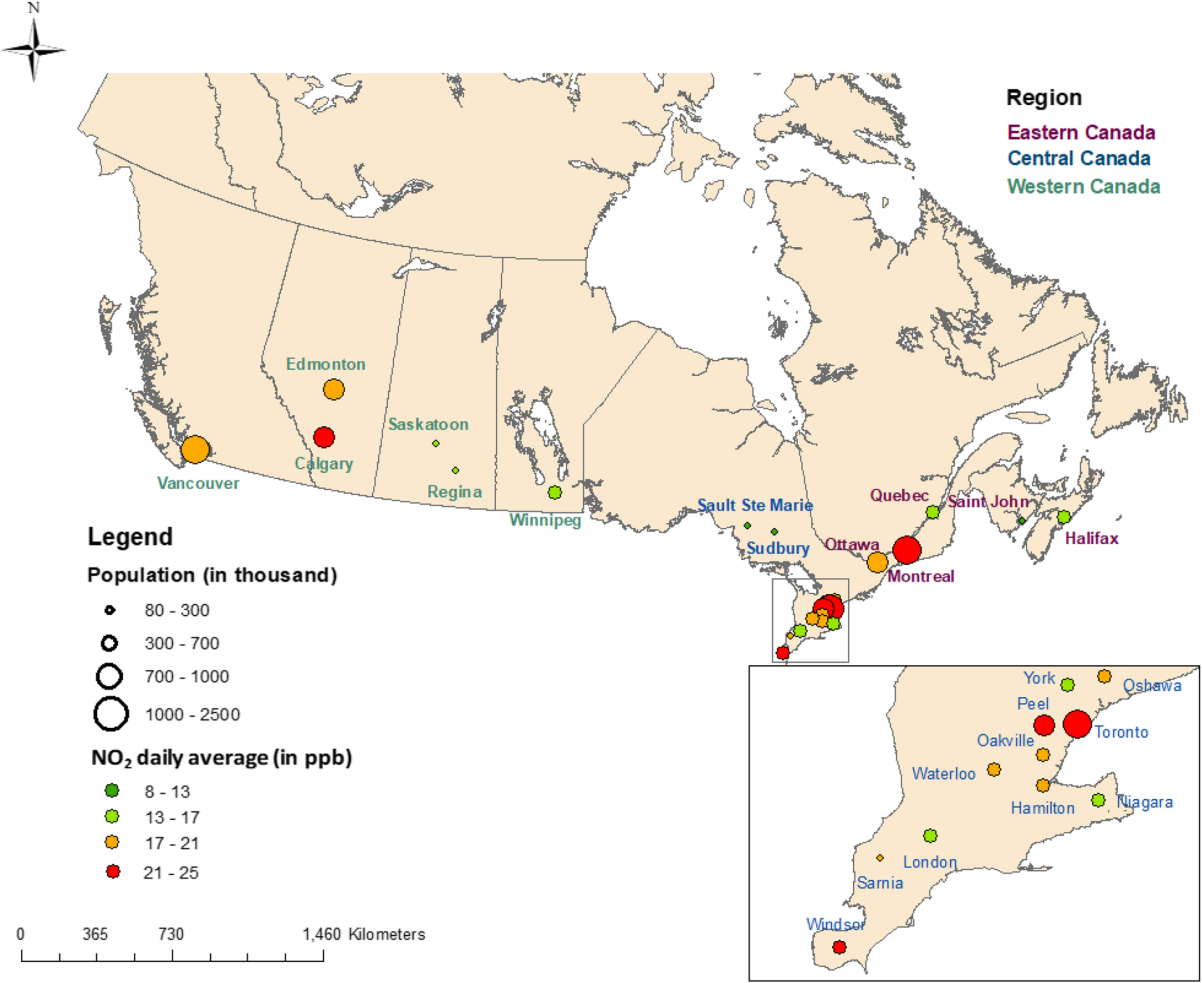
A map of 24 census divisions: (1) population size, circle size in 4-scale; (2) NO_2_ concentration level, color in 4-scale circle color from green to red for higher concentration; and (3) three regions in coloured text (purple for Eastern, blue for Central, and teal for Western Canada)

We studied acute hospitalization associated with short-term exposure to ambient NO_2_ concentrations, by estimating, separately, 0- up to 6-day lagged effects of NO_2_ exposure on hospital admissions. Based on the criterion of having a reasonably complete time series of NO_2_ and sufficient daily hospitalizations, 24 Canadian cities were selected for this study (Fig. 1). We conducted an ecological time-series study in the 24 cities, covering about 52% of the Canadian population, over 17 years. The study period was determined solely by data availability. The geographic boundaries of each city were defined by Census Division (CD) based on the four census years 1996, 2001, 2006 and 2011 of the study period [17]. The CD is an intermediate geographical unit between the province and municipality level, consisting of counties or their equivalents. We selected the CD as the geographical unit for two reasons: (1) their geographic boundaries were stable over time, and (2) they had a reasonable population, which was critical for sufficient daily counts of hospitalization.

Using stratified models, we considered five factors for regional differences: cause, season, age, sex, and temporal trend. Overly specific causes were avoided since reasonably large daily hospitalization counts were required. We selected three broad causes (all-cause, circulatory, and respiratory) along with three specific causes related to the circulatory system, and two with the respiratory system (see the next subsection on Data). We considered two seasons: warm between April and September versus cold between October and March. For age, we considered two groups: non-senior (1 to 65 years old, inclusive), and senior (≥ 66 years old). For sex, we considered female and male only based on genotypic sex classification. Finally, for trend we considered annual variations in hospitalization risk, applying a dynamic model approach during the study time period (see the next subsection on Hierarchical Models). Dynamic models allow time-varying risks, whereas static models assume a constant risk over time. The trend is expected to reveal changes in the impact of NO_2_ on hospitalizations over time, which may depend on socio-demographic and environmental changes, health care systems, access to medical facilities, etc.

### Data

We collected three types of data from Canadian cities over the study period from 1996–2012: daily counts of cause-specific hospitalizations, hourly NO_2_ concentrations, and confounders (e.g. daily temperature). Since the time resolution in this study is one day, only confounders varying day-to-day were considered.

We selected CDs with data for at least 80% of the days in each year (i.e. 146 days for seasonal estimates and 292 days for year-round estimates). For regional risk estimates, we classified the 24 CDs into 3 regions based on geographical location: Eastern, Central and Western Canada (see the next subsection on Hierarchical Models).

The source of hospitalization was the Discharge Abstract Database (DAD) provided by the Canadian Institute for Health Information (CIHI). We extracted only unplanned admissions to the hospital (i.e., unplanned surgeries, heart attacks, etc.) for this study, including urgent admission not through emergency because a patient may be transferred from another facility on an urgent (i.e., “unplanned”) basis, but excluding emergency visits, which were captured in the National Ambulatory Care Reporting System (NACRS). For multiple admissions, we separated repeated hospitalizations, because our focus was short-term exposure. We used daily counts of hospitalizations, so the individuals were counted for each day of their multiple hospitalizations.

Hospitalization data were extracted only when the CD of residence corresponded with the CD of hospital admission, which was required to assign NO_2_ exposures. Based on the International Classification of Diseases (ICD)−10 [18], the hospitalization data included only admissions from internal causes (ICD-10 code A00-R99), excluding those related to external causes such as injuries. We collected hospitalization data from eight specific causes: (1) all-cause (ICD-10, A00-R99); (2) circulatory system (I00-I99); (3) respiratory system (J00-J99); (4) ischemic heart disease (IHD, I20-I25); (5) other heart disease (OHD, I30-I52); (6) cerebrovascular disease (CEV, I60-I69); (7) influenza/pneumonia (InfPn, J09-J18); and (8) chronic lower respiratory diseases (CLRD, J40-J47). For the ICD code there were changes from ICD-9 to ICD-10 during 2001–2004, varying over cities. To keep consistency in data extraction during the study period (1996–2012), we used ICD-10 code and a conversion chart from ICD-10 to ICD-9 for earlier years of the study period. The conversion chart was accurate for two reasons: it was provided by the Canadian Institute for Health Information (CIHI), the hospitalization data provider; and there was no drastic change in annual counts of the abovementioned health outcomes around the time of the conversion.

Hourly NO_2_ concentrations were obtained from the National Air Pollution Surveillance (NAPS) Program [19] operated by Environment and Climate Change Canada. For each NAPS monitoring station, the daily average concentration was calculated if at least 18 hourly concentrations were available, and recorded as missing otherwise. For each CD, daily average concentrations were in turn averaged if two or more stations were present within the CD. To estimate the short-term effect of NO_2_ on hospitalizations, we examined 0- up to 6-day lagged effects, respectively.

Three potential confounding variables to the NO_2_-hospitalization association were considered: time, temperature, and indicators for day of the week. Calendar time controls for temporal and seasonal variations, daily temperature controls for short-term effects of weather on daily hospitalization, and day of the week accounts for weekly cycles in hospitalization. To account for weather effects, daily mean temperature data were obtained from the National Climate Data and Information Archive of Environment and Climate Change Canada [20]. Among the 24 CDs, 8 CDs had no hourly relative humidity or dew point temperature data for 10 or more years. Modelling the remaining 16 CDs with and without relative humidity and dew point indicated negligible differences. Relative humidity and dew point temperature were thus not included as covariates in our model.

### Hierarchical Models (city-specific, regional and national risks)

We assumed daily counts of hospitalization (response) are associated with ambient NO_2_ (main predictor) and day of the week linearly, and with time and temperature in a non-linear relationship through a *generalized additive model* (Eq. 1). We first estimated CD-specific risk of acute hospitalization associated with short-term exposure to NO_2_ concentrations, applying an *over-dispersed Poisson model*, and then estimated regional and national risks using a *Bayesian hierarchical model* for each cause, season, age, and sex.

1 $$\text{log}\left(E\left[{Y}_{ijk}\left(t\right)\right]\right)={\beta }_{0ijk}+{\beta }_{1ijk}*{x}_{ij}\left(t-l\right)+{f}_{ij}\left(t\right)+{g}_{ij}\left(temp\left(t\right)\right)+{DOW}_{i}\left(t\right),$$

where ${Y}_{ijk}\left(t\right)$ is daily counts (the response variables), ${x}_{ij}(t-l)$ is $l$-day lagged daily NO_2_ concentrations (the main predictor) for $l=0,\dots 6$, and ${DOW}_{i}\left(t\right)$ is the day of week (a confounder) for region *i* = *1, 2*, *3,* CD *j* = *1, 2,…, 24*, age group or sex *k* = *1, 2*, and day $t=1, 2,\dots ,T$. For short-term exposure, we examined single-day exposures within a week, i.e. 0- up to 6-day prior to hospitalization. Two more confounders, ${f}_{ij}\left(t\right)$ and ${g}_{ij}\left(temp(t)\right),$ are smoothing functions for calendar time and temperature to account for seasonal and immediate temperature effects on health outcomes. For the time smoother, ${f}_{ij}\left(t\right)$, we applied Discrete Prolate Spheroidal (Slepian) Sequences among various smoothers to separate short-term (within a 2-week time span) from long-term effects of air pollution [21]. The Slepian smoothing (a non-standard method) is employed since it can remove long-term effect such as trend and seasonal associations, and thus reduce bias in estimates. For the temperature smoother, we applied a natural cubic spline with 3 degrees of freedom based on a U-shape relation between temperature and health outcome, accounting for immediate temperature effect on health outcomes. The ${\beta }_{1ijk}$ represents NO_2_-related CD-specific risk of hospitalization, which is to be estimated. For the Bayesian hierarchical model for national risk, we used non-informative prior distributions using 5 chains with 10,000 iterations (excluding 1,000 burn-in) each, and reported national associations with 95% posterior intervals, i.e. highest density intervals, as the posterior distributions were all unimodal. More detailed model descriptions for the hierarchical models can be found elsewhere [1, 16].

For regional risk estimates, we clustered the cities into three regions based on their geographical locations and intrinsic link (eg: inter-dependence of services): Eastern, Central, and Western Canada. The Eastern region consisted of Ottawa and 4 cities in the provinces east of Ontario, while the Western region was comprised of 6 cities in the provinces west of Ontario. The Central region included 13 geographically close cities all located in Ontario (Fig. 1).

- Eastern region (5 CDs): Halifax, Saint John, Quebec, Montreal, and Ottawa.
- Central region, (13 CDs): Oshawa, York, Toronto, Peel, Oakville, Hamilton, Niagara, Waterloo, Windsor, Sarnia, London, Sudbury, and Sault Ste. Marie.
- Western region (6 CDs): Winnipeg, Regina, Saskatoon, Calgary, Edmonton, and Vancouver.

Using hierarchical models, we compared national and regional risks (the highest single-day lagged effect), and investigated regional differences by cause, season, age group and sex. We applied multi-year (7-years, between 2002–2012) estimators [22, 23] to report annual risk estimates for temporal trends in each region. To detect linear trends in these annual risk estimates we used Sen’s test [24]. All estimates were calculated using R statistical software (version 4.2.1) [25].

## Results

Figure 1 displays the included CDs, indicating population size and NO_2_ concentration levels in 4-scale and the three regions. Populations were stable between the Western (30%), Central (48%), and Eastern (24%) regions (Supplementary Materials: Table S1). The age-sex composition of the study population is summarised by region in Table S2. The senior population increased in growth by 42% compared to 24% in the overall study population, indicating an aging Canadian population. The Central region showed the highest increase in seniors (49%), driven primarily by York (162%) and Peel (132%) (Table S2). On the other hand, the regional sex ratios were stable over time (females, 50%−51%), but unbalanced in the senior population (more females, 56–61%).

Table 1 summarizes annual ratios of hospitalizations by cause (all-cause, circulatory, respiratory), with ratios for sex (female), and season (warm), and overall regional ratios. Tables S3W, S3C, and S3E provide the same information for each region (though regional incidence is reported as a rate). All-cause hospitalizations occurred at a rate of 6,836 per 100,000, decreasing from 8,337 (1996) to 6,362 (2012), with females 61%. Circulatory hospitalizations accounted for 15% of all-cause hospitalizations, compared to 9% for respiratory hospitalizations. Sex differences were observed for circulatory hospitalization, whereas respiratory hospitalization varied by season. Overall, females were hospitalized more frequently for all-cause hospitalization (61% vs 39%) and less so for circulatory (43% vs 57%) and respiratory (48% vs 52%) causes. There were also seasonal differences by cause that were common to the overall study population and each region such that more respiratory hospitalizations were observed during the cold season (57% vs 43%). However, this seasonal pattern was not seen for circulatory hospitalization (51% vs 49%). There was a noticeable regional difference in all-cause hospitalization rate, which was higher in the Western region (7,236) than the Eastern (6,622) and Central (6,687) regions. However, there were no differences in regional circulatory or respiratory hospitalization rates.

**Table 1:** Annual ratio of hospitalization counts to population by cause, sex and season from 1996–2012

	**All non-accidental (ICD-10, A00-R99)**				**Circulatory (ICD-10, I00-I99)**				**Respiratory (ICD-10, J00-J99)**			
Year	Count	% ratio^a^	% female^b^	% in warm^c^	% ratio^a^	% of all causes^d^	% female^b^	% in warm^c^	% ratio^a^	% of all causes^d^	% female^b^	% in warm^c^
1996	2,840,181	9.6	59	49	1.5	12	44	49	1.0	10	47	43
1997	2,731,223	9.1	59	50	1.5	12	43	50	0.9	10	47	43
1998	2,694,759	8.9	59	49	1.4	12	43	49	1.0	11	48	41
1999	2,667,100	8.8	59	49	1.4	12	43	49	1.0	11	48	39
2000	2,604,227	8.5	59	49	1.4	12	43	49	0.9	10	47	42
2001	2,532,790	8.2	59	49	1.3	12	43	49	0.8	10	47	43
2002	2,447,756	7.8	59	49	1.3	12	43	49	0.8	10	47	44
2003	2,416,859	7.6	58	49	1.2	11	42	49	0.8	10	47	43
2004	2,426,161	7.6	58	49	1.2	11	42	49	0.7	10	48	44
2005	2,027,956	6.3	58	47	0.9	11	42	46	0.7	10	48	41
2006	1,849,774	5.7	58	49	0.8	11	42	49	0.5	9	47	45
2007	1,831,969	5.6	58	50	0.8	10	42	49	0.5	9	47	44
2008	1,829,618	5.5	58	50	0.8	10	41	49	0.5	9	48	45
2009	1,840,600	5.5	58	50	0.8	10	41	50	0.5	9	48	43
2010	1,855,603	5.5	58	50	0.8	10	42	50	0.5	9	48	46
2011	1,889,247	5.5	58	50	0.8	10	41	50	0.5	9	48	44
2012	1,922,830	5.5	58	50	0.8	10	41	49	0.5	9	48	44
**Eastern**	561,078	5.7	57	48	1.0	12	42	48	0.6	11	48	42
**Central**	896,360	7.3	58	50	1.2	11	42	49	0.7	9	47	43
**Western**	801,895	8.3	59	50	1.1	11	42	49	0.8	10	47	44
**Overall**	2,259,333	**7.1**	**58**	**49**	**1.1**	**11**	**42**	**49**	**0.7**	**10**	**47**	**43**

^a^(Study all hospitalization counts/ Study population) *100

^b^(hospitalization counts for females (≥ 1 year old) / hospitalization counts for both sexes (≥ 1 year old)) *100. This includes admissions for childbirth

^c^(hospitalization counts in the warm season (April-September) / year-round (January-December) counts) *100

^d^(specific-cause hospitalization counts/ Study all non-accidental counts) *100, where Study all non-accidental counts are in the 2nd column

Table 2 summarizes five annual cause-specific hospitalization rates (IHD, OHD, CEV, CLRD, and InfPn) with ratios for sex (female) and season (warm), and overall regional ratios. Tables S4W, S4C, and S4E provide the same information for Eastern, Central, and Western regions, respectively. Among the five aforementioned specific causes, the overall hospitalization rate was highest for IHD (418) and lowest for CEV (151). There was a difference by sex for IHD (males, 66%) and a seasonal difference for InfPn (cold, 59%). Unlike the general cause hospitalizations (all-cause, circulatory, and respiratory), differences were more apparent in the regional population ratios. In the Central region, cause-specific hospitalization rates were higher for circulatory (IHD, OHD and CEV) but lower for respiratory (CLRD, and InfPn) than the other regions. In contrast, there was little difference regionally in sex and seasonal ratios for the five cause-specific hospitalizations.

**Table 2:** Annual ratio of specific-cause hospitalization counts to population from 1996–2012

Year	**Ischemic Heart Disease (IHD, ICD-10, I20-I25)**			**Other forms of Heart Diseases (OHD, ICD-10, I30-I52)**			**Cerebrovascular Diseases (CEV, ICD-10, I60-I69)**			**Chronic Lower Respiratory Diseases (CLRD, ICD-10, J40-J47)**			**Influenza and Pneumonia (InfPn, ICD-10, J09-J18)**		
	% ratio^a^	% female^b^	% in warm^c^	% ratio^a^	% female^b^	% in warm^c^	% ratio^a^	% female^b^	% in warm^c^	% ratio^a^	% female^b^	% in warm^c^	% ratio^a^	% female^b^	% in warm^c^
1996	0.45	38	49	0.33	49	49	0.19	51	50	0.24	51	45	0.19	48	41
1997	0.44	38	50	0.33	49	49	0.18	51	49	0.22	50	47	0.18	48	40
1998	0.43	37	49	0.33	49	49	0.18	51	50	0.23	51	41	0.21	48	38
1999	0.43	37	49	0.31	49	48	0.17	51	49	0.24	51	41	0.22	49	35
2000	0.43	37	49	0.30	49	49	0.17	52	50	0.20	51	45	0.19	49	40
2001	0.41	36	49	0.29	49	48	0.15	52	49	0.19	51	46	0.18	49	43
2002	0.40	36	49	0.28	49	49	0.14	51	48	0.19	50	46	0.16	48	41
2003	0.38	35	48	0.27	48	48	0.13	51	49	0.19	50	45	0.15	49	40
2004	0.37	35	49	0.28	49	49	0.13	50	50	0.20	50	45	0.14	49	41
2005	0.34	35	49	0.28	48	49	0.13	50	49	0.22	50	45	0.15	50	41
2006	0.32	35	49	0.27	49	48	0.12	49	49	0.20	50	47	0.13	49	42
2007	0.30	33	48	0.27	48	49	0.12	49	49	0.19	50	45	0.12	49	44
2008	0.29	33	48	0.27	48	49	0.12	49	49	0.19	50	47	0.13	50	44
2009	0.27	33	49	0.27	49	49	0.11	50	49	0.18	51	45	0.15	50	40
2010	0.27	33	49	0.28	49	49	0.11	49	50	0.18	51	47	0.13	50	43
2011	0.25	33	49	0.28	49	49	0.11	49	49	0.18	51	46	0.14	50	42
2012	0.25	33	49	0.29	48	49	0.11	49	50	0.19	51	45	0.14	50	41
**Eastern**	0.22	36	49	0.17	49	48	0.09	51	49	0.12	54	44	0.09	51	41
**Central**	0.60	37	49	0.49	49	49	0.22	50	49	0.31	50	45	0.26	49	41
**Western**	0.18	33	49	0.15	49	49	0.07	50	50	0.12	50	48	0.09	49	43
**Overall**	**0.35**	**35**	**49**	**0.29**	**49**	**49**	**0.14**	**50**	**49**	**0.20**	**50**	**45**	**0.16**	**49**	**41**

^a^(Study specific hospitalization counts/ Study population) *100

^b^(hospitalization counts for females (≥ 1 year old) / hospitalization counts for both sexes (≥ 1 year old)) *100

^c^(hospitalization counts in the warm season (April-September) / year-round (January-December) counts) *100

Table 3 summarizes the number of NAPS ground monitoring stations and annual average NO_2_ concentration and temperature by season for each CD and region. There were a total of 114 NAPS stations distributed in the Central (43 stations, 38%), Western (39 stations, 34%), and Eastern (32 stations, 28%) regions. Compared to their population ratios, i.e. Central (48%), Western (30%), and Eastern (24%), the Western region had relatively more NAPS stations than the other regions. The number of NAPS stations appeared proportional to geographical area rather than population size. At the CD level, the Western region had the highest variation in number of NAPS stations: for example, 21 NAPS stations for Vancouver and only 1 station for Saskatoon.

**Table 3:** Annual average concentration of NO_2_ and temperature by season from 1996–2012

City^a^	Number of NAPS	NO_2_^b^ (SD^c^)		Temperature^d^ (SD^c^)	
		Warm^e^	Cold^e^	Warm^e^	Cold^e^
Halifax	5	11 (7)	13 (7)	13 (6)	1 (6)
Saint John	4	7 (5)	7 (6)	12 (5)	0 (7)
Quebec City	6	9 (4)	15 (8)	14 (6)	−4 (8)
Montreal	14	14 (6)	19 (8)	16 (6)	−2 (8)
Ottawa	3	11 (8)	16 (9)	16 (6)	−2 (8)
Durham	2	12 (9)	14 (9)	16 (6)	0 (7)
York	2	8 (4)	12 (7)	16 (6)	0 (8)
Toronto	11	20 (8)	23 (8)	17 (6)	2 (7)
Peel	4	15 (8)	19 (9)	17 (6)	1 (7)
Halton	3	14 (6)	16 (7)	17 (6)	1 (7)
Hamilton	4	15 (8)	18 (8)	16 (6)	1 (7)
Niagara	2	11 (6)	15 (7)	17 (6)	2 (7)
Waterloo	2	9 (5)	14 (8)	16 (6)	0 (7)
Windsor	3	16 (7)	20 (8)	18 (6)	2 (7)
Sarnia	2	12 (7)	14 (8)	17 (6)	1 (7)
London	3	11 (5)	15 (8)	16 (6)	1 (7)
Sudbury	2	6 (3)	9 (5)	14 (7)	−5 (9)
Sault Ste. Marie	3	6 (4)	10 (6)	13 (6)	−4 (8)
Winnipeg	2	9 (4)	15 (7)	15 (7)	−6 (10)
Regina	2	10 (4)	14 (6)	13 (6)	−8 (10)
Saskatoon	1	9 (4)	14 (6)	13 (6)	−8 (10)
Calgary	4	15 (6)	26 (9)	11 (6)	−4 (8)
Edmonton	9	9 (4)	16 (8)	12 (6)	−5 (9)
Vancouver	21	13 (4)	17 (5)	15 (4)	6 (4)
**Eastern**	32^f^	11 (6)	14 (9)	15 (6)	−1 (8)
**Central**	43^f^	12 (7)	15 (8)	16 (6)	0 (8)
**Western**	39^f^	11 (5)	17 (8)	13 (6)	−4 (10)
**Combined**	**114**^f^	**11 (7)**	**16 (8)**	**15 (6)**	**−1 (9)**

^a^Cities are ordered geographically from east to west

^b^NO_2_ concentrations are calculated using the imputed 24-h daily average in ppb

^c^Standard deviation of annual values for 17 years, 1996–2012

^d^Temperatures are calculated using 24-h daily average in °C

^e^Warm (April to September); Cold (October to March)

^f^Number of all NAPS within region or nation

The average NO_2_ concentration across the 24 CDs from 1996 – 2012 was 14 ppb (SD = 8). Annual average NO_2_ concentrations were higher in the cold season for all CDs and regions, with the exception of Saint John that was the same in both warm and cold temperatures. The highly populated Central region had the highest NO_2_ concentrations in the warm season whereas the Western region had the highest NO_2_ concentrations in the cold season. Eastern Canada, the least populated of the three regions, had the lowest NO_2_ concentrations for both seasons. In particular, higher NO_2_ concentrations (≥ 20 ppb) were observed in four CDs (Toronto, Peel, Hamilton, and Windsor) in the Central region, and two CDs in both the East (Montreal and Ottawa) and West (Calgary and Edmonton). These concentrations can be interpreted with respect to the Canadian Ambient Air Quality Standards (CAAQS) targets for NO_2_, which are set at 17.0 ppb for 2020 and decrease to 12.0 ppb in 2025 [26].

Regionally there was little difference in annual average temperatures, which overall were 15 °C during the warm season and −1 °C for the cold season. The Western region had lower temperatures in both seasons compared to the other regions.

**Fig. 2:**
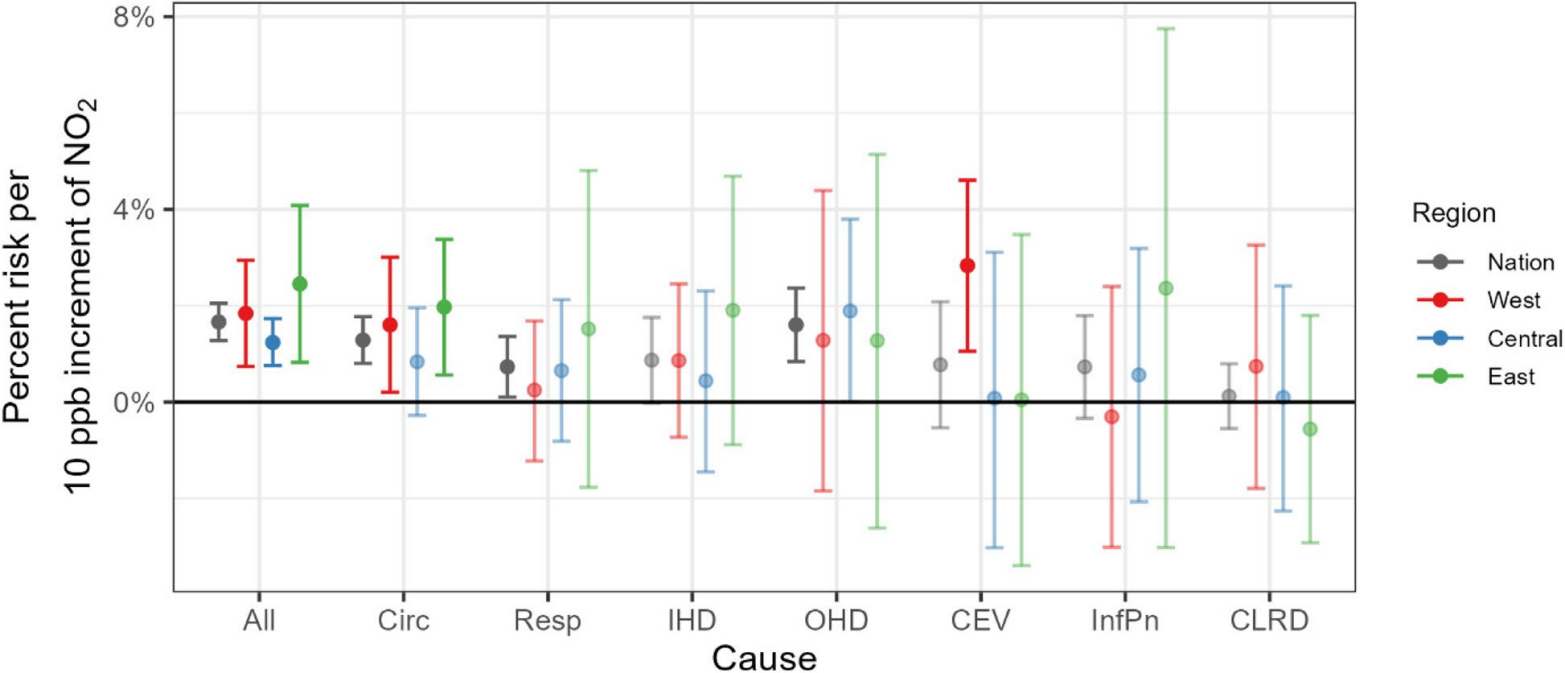
Comparison of estimated year-round regional associations, with 95% credible intervals, between NO_2_ and cause-specific hospitalization. Note that dark and light color indicates statistically significant and insignificant estimates

Figure 2 displays year-round risk (%) of acute hospitalizations by cause at the national and regional levels (per 10-ppb increase in NO_2_ concentrations) for the whole study population (both sexes aged ≥ 1 year). Overall we found limited regional differences in the effect of NO_2_. For example, all-cause hospitalization regional risk estimates were all significant and comparable in effect size and direction of NO_2_: 1.8% (95% credible interval, 0.7%−2.9%) for the Western region, 1.2% (0.8%−1.7%) for the Central region, and 2.5% (0.8%−4.1%) for the Eastern region. However, some regional differences were found with respect to uncertainty size and cause. First, greater uncertainty was apparent for the Eastern and Western regions, and respiratory hospitalizations. This can be explained by population size and the range of daily counts. Second, we found regional differences for circulatory but not respiratory hospitalizations. The Eastern and Western regions were at a significantly higher risk of circulatory hospitalization but not the Central region: 1.6% (0.2%−3.0%) for the Western region, 2.0% (0.6%−3.4%) for the Eastern region, and 0.8% (−0.3%−2.0%) for the Central region. In particular, the Western region was at a higher risk of CEV-related hospitalization than the other regions: 2.8% (1.1%−4.6%) for the Western region, 0.1% (−3.0%−3.1%) for the Central region, and 0.0% (−3.4%−3.5%) for the Eastern region. We found no regional differences for all other causes.

**Fig. 3:**
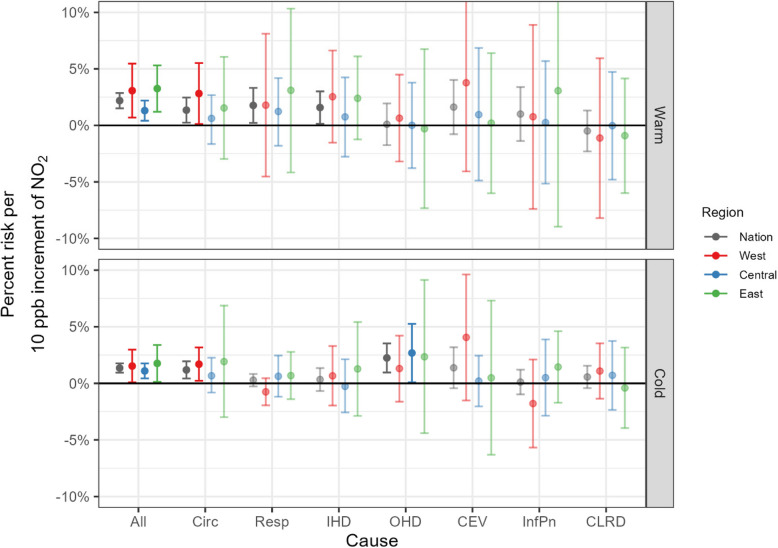
Comparison of estimated regional associations, with 95% credible intervals, between NO_2_ and cause-specific hospitalization, by season (warm: April-September; cold: October–March). Note that dark and light color indicates statistically significant and insignificant estimates

Figure 3 displays regional differences in acute hospitalizations associated with exposures to ambient NO_2_ by season for the whole study population. There was higher variability and more uncertainty for warm season risk estimates. We found that regional risks differed by season only for OHD hospitalization, which had significant estimates for the cold season: 1.3% (−1.6%−4.2%) for the Western region, 2.7% (0.1%−5.3%) for the Central region, and 2.3% (−4.4%−9.1%) for the Eastern region.

**Fig. 4:**
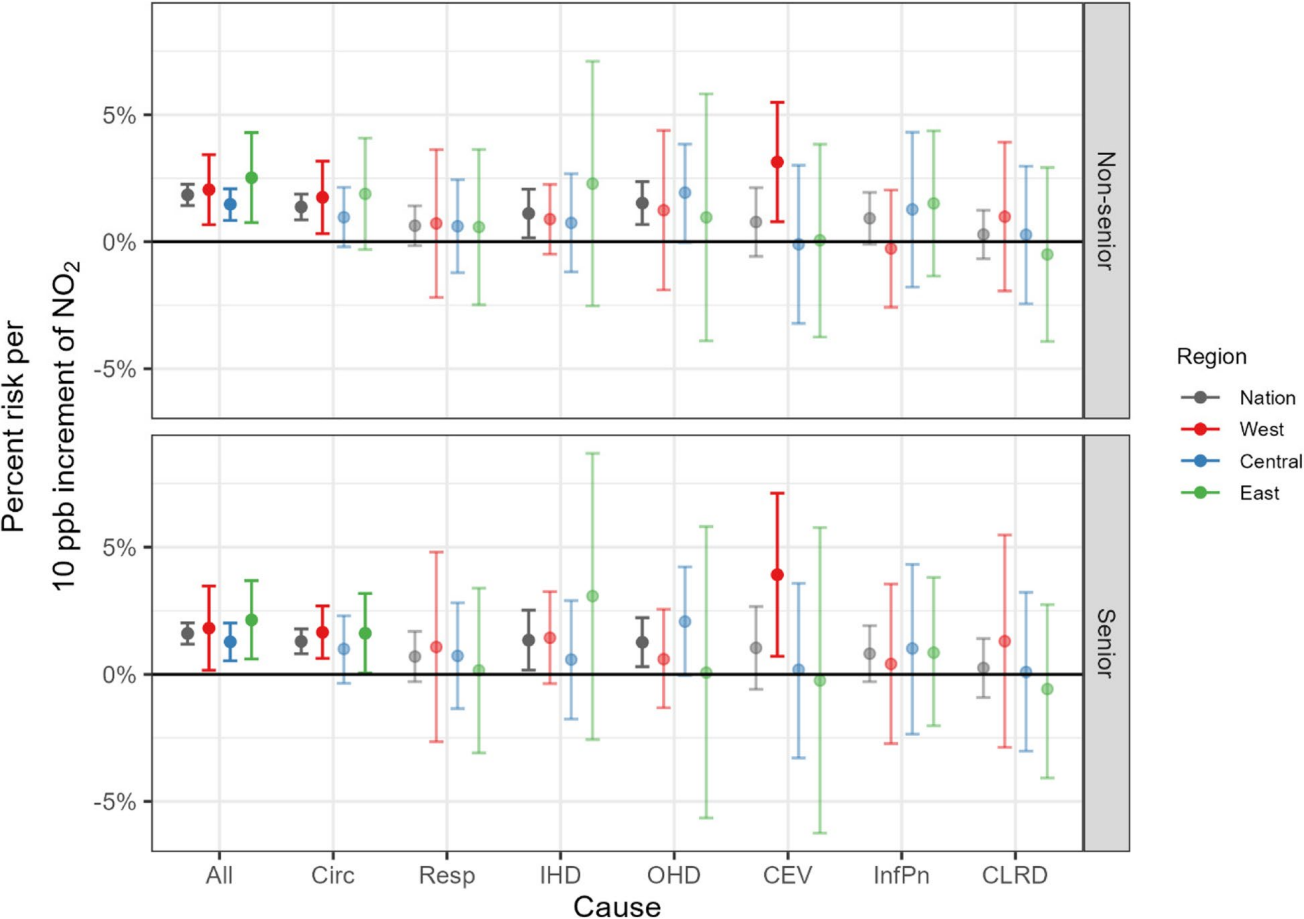
Comparison of estimated year-round regional associations, with 95% credible intervals, between NO_2_ and cause-specific hospitalization, by age: non-seniors (aged 1 to 65 years, inclusive) and seniors (≥66 years) . Note that dark and light color indicates statistically significant and insignificant estimates

Figure 4 displays year-round regional differences in acute hospitalizations associated with exposures to ambient NO_2_ by age group. Overall, we found larger uncertainty for seniors, and little change in significance by age regionally. Only circulatory hospitalization exhibited a regional difference by age, which had insignificant risk for non-seniors but became significant for seniors in the West, 1.7% (0.6%−2.7%).

**Fig. 5:**
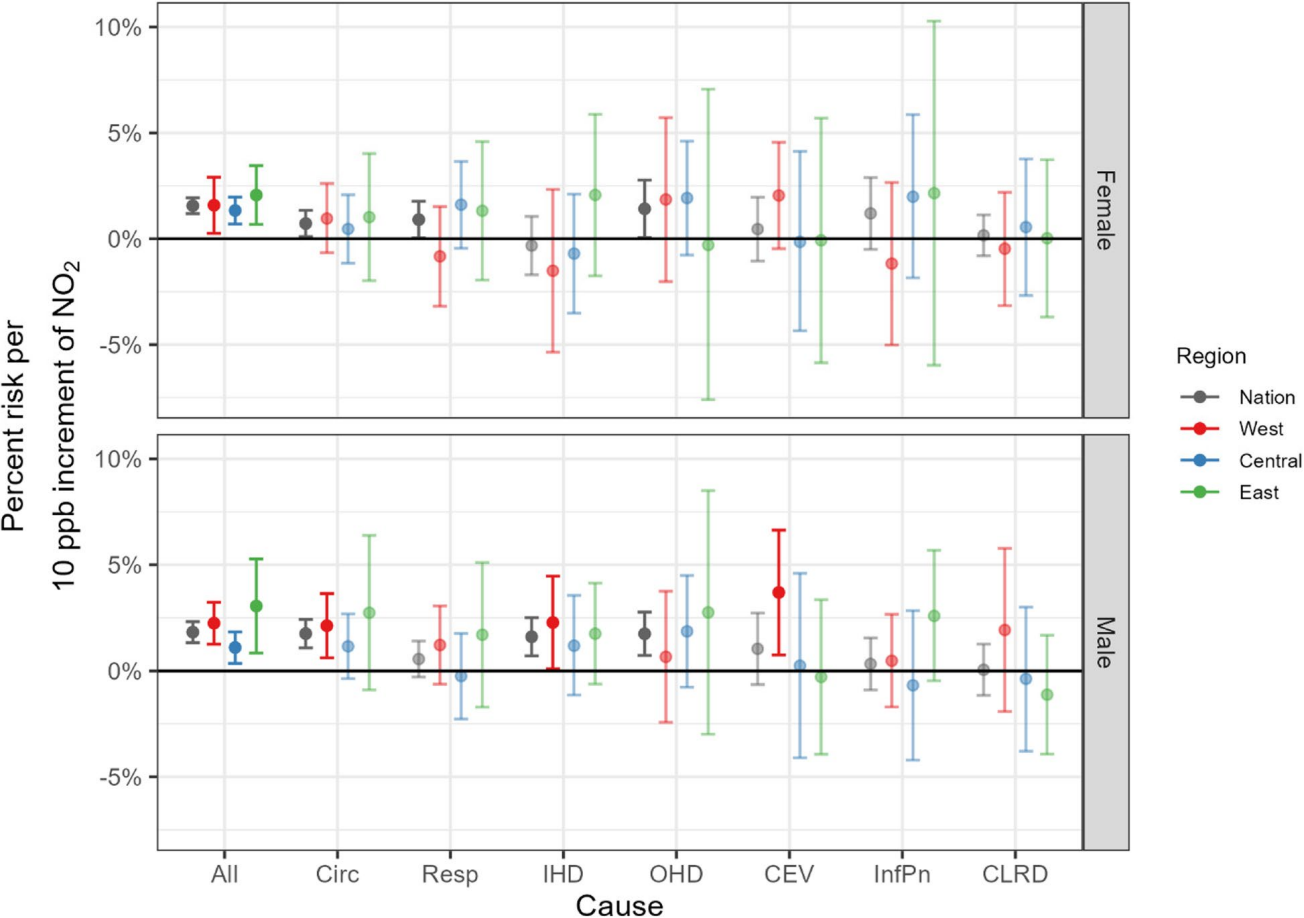
Comparison of estimated year-round regional associations, with 95% credible intervals, between NO_2_ and cause-specific hospitalization, by biological sex: females (top) and males (bottom). Note that dark and light color indicates statistically significant and insignificant estimates

Figure 5 displays year-round regional differences in acute hospitalizations associated with exposures to ambient NO_2_ by sex. There was higher variability and larger uncertainty in risk estimates for females overall. We found regional effects differed by sex for circulatory but not respiratory hospitalization. Only the Western region had significant risk of circulatory hospitalization for males, 2.1% (0.6%−3.6%), compared to 1.2% (−0.4%−2.7%) in the Central region, and 2.7% (−0.9%−6.4%) in the Eastern region. Similarly, risks were only significant for males in the Western region for IHD and CEV: for IHD, 2.3% (0.1%−4.5%) for the Western region, 1.2% (−1.1%−3.6%) for the Central region, and 1.8% (−0.6%−4.1%) for the Eastern region; and for CEV, 3.7% (0.8%−6.6%) for the Western region, 0.2% (−4.1%−4.6%) for the Central region, and −0.3% (−3.9%−3.4%) for the Eastern region.

**Fig. 6:**
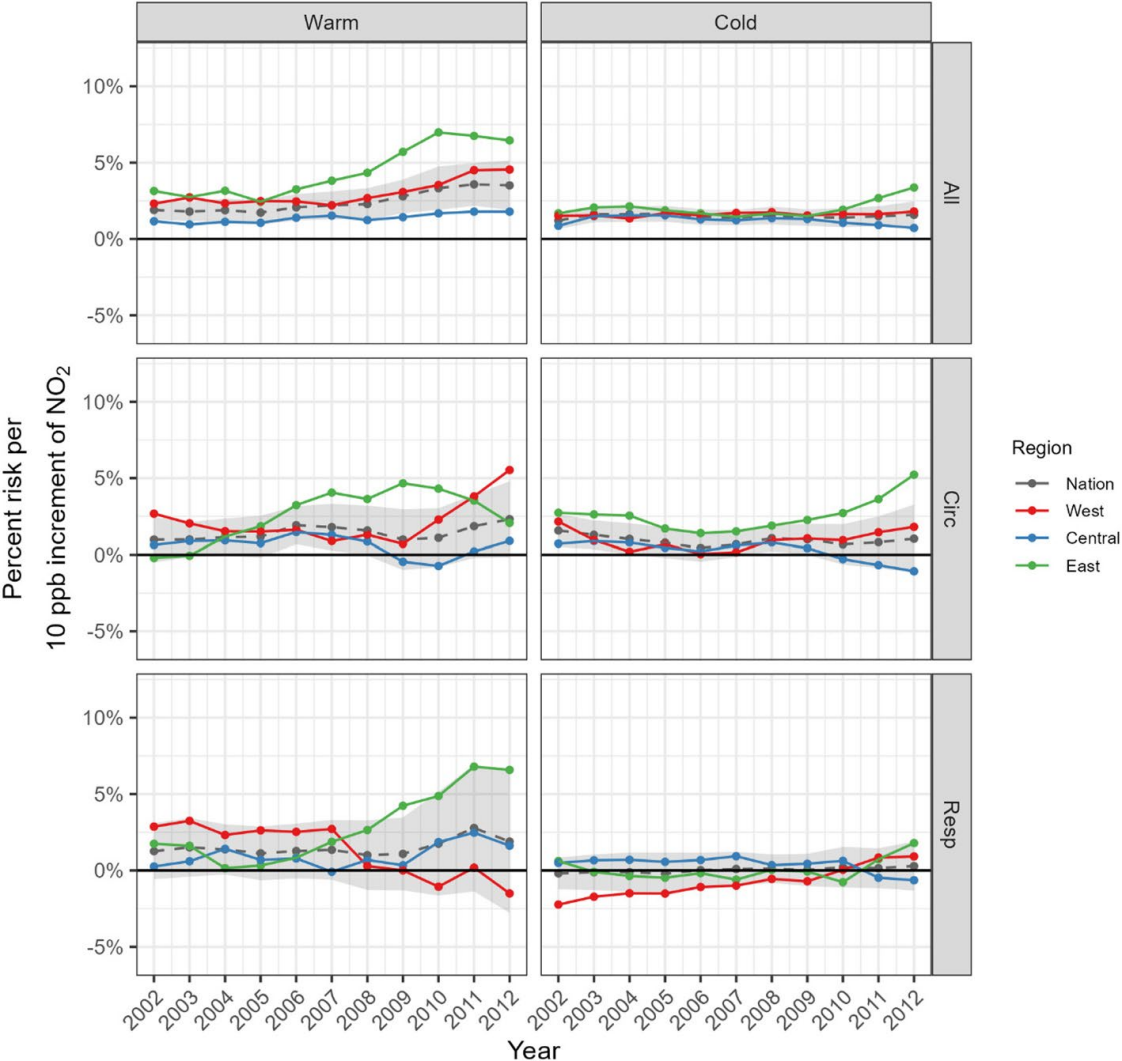
Trends in estimated associations between NO_2_ and hospitalizations by season (warm and cold season in column) and cause of hospitalization (all-cause, circulatory and respiratory in row). A 95% credible interval for the estimated national risk is shown in grey

Figure 6 displays trends in national and regional risks of NO_2_-related acute hospitalizations for three causes (rows) and two seasons (columns). The shaded areas indicate 95% credible intervals of national risk estimates as a reference range. Overall, we found (1) more visible regional differences for circulatory hospitalization and the warm season, (2) that the Eastern region was close to or above the upper level of the reference range, whereas the other regions were more often within the reference range, and (3) the Central region had relatively stable risks of acute hospitalizations over time. In both seasons there was a noticeable increase in the regional differences over time, particularly in the latter years. Only all-cause hospitalization during the warm season exhibited a consistent upward trend.

For specific hospitalization causes, regional differences are visible for IHD (warm season) and CEV (cold season) as shown in Supplementary Materials **(**Figures S1 and S2). In contrast, there were no increasing or decreasing trends in regional differences for OHD, InfPn, or CLRD. In addition, the Eastern region showed more varied trends, fluctuating for most specific hospitalization causes. Figures S3 and S4 display regional trends for cause and season, by age and sex. For cold season circulatory hospitalization there was an increasing trend for males in the Eastern region, a decreasing trend for all groups except females in the Central region, but no trend in the Western region. For warm season respiratory hospitalization there were increasing trends in the Central and Eastern regions for males and seniors, whereas in the West there was a decreasing trend for these groups.

## Discussion

To our knowledge, this study provides the first epidemiological evidence of regional differences in association with cause-specific hospitalization and short-term exposure to ambient NO_2_ across Canada. The study findings indicate that adverse health effects of NO_2_ on acute hospitalization depend on cause and season rather than geographical regional differences. For example, all-cause hospitalization risk estimates were similar between national and regional groups indicating limited regional differences but risk estimates were statistically significant for circulatory hospitalization during the cold season and respiratory hospitalization during the warm season. We also found that the association between NO_2_ and respiratory hospitalizations was less stable over time than for circulatory hospitalizations, that risks were higher during the warm season, and there was an increasing trend for respiratory hospitalization but not circulatory hospitalizations. Among the three regions, only the Western region showed a higher risk of circulatory (not respiratory) hospitalization. We found no increasing or decreasing trends in regional risks for year-round circulatory or respiratory hospitalization. However, increasing trends in regional differences for the warm season were detected, which is consistent with the increasing regional heterogeneities for NO_2_-related mortality [1].

Nitrogen dioxide is rapidly formed from NO emissions which come mainly from transportation (road vehicles 35% and other transportation 35%) [27]. NO_2_ also reacts with sunlight to convert back to NO, produce ozone, and contribute to the formation of PM_2.5_ [28]. Given these sources and atmospheric reactions, NO_2_ concentrations and their adverse health effects are expected to vary spatially and temporally. For example, economic activity and climate vary geographically, and electrified vehicles (non-tailpipe emissions only) are becoming popular over time. The Canadian Environmental Sustainability Indicators (CESI) quantified variations in annual average NO_2_ concentrations along with the 10th and 90th percentiles in Canada from 2005 to 2019 and found regional differences among the five regions (Atlantic, Southern Quebec, Southern Ontario, Prairies and northern Ontario, and British Columbia) [29]. These differences could lead to regional variations in the adverse health effect of NO_2_ despite the fact that the CESI used five regions based on 179 NAPS stations, whereas this study used three regions based on 24 cities with 112 NAPS stations; however, only limited statistically significant regional differences were observed in this study. Nonetheless, we need to understand the possible causes of these regional differences.

This study reports regional differences in NO_2_-related circulatory health outcomes but not respiratory outcomes. Considering the biological mechanisms in cardio-respiratory function, our regional differences may relate to age or sex composition of each region, rather than geographical location. Previous studies on the effect of NO_2_ by age on mortality and hospitalization have been inconsistent. Among seniors aged 65 years and older, associations were observed for cardiovascular mortality in Montreal [14] but not for hospitalization due to cardiovascular disease in Denver [12]. In contrast, a study of 10 Canadian cities found stronger associations among individuals aged < 60 years compared with those aged 60–79, and no association among those aged 80–89 [9]. Similarly, there were higher risks of respiratory hospitalization for the base group (> 1 year of age) than seniors (> 65 years) during the warm season [13]. In Sao Paulo, Brazil the percent change in mortality associated with an IQR increase in NO_2_ was higher among those aged 65–74 and 75 + than those aged 35–64 years; however, differences were not apparent for respiratory or cardiovascular mortality [30]. Age was not observed to have a significant effect on the association between NO_2_ and mortality [5]. The results reported herein show that the regional differences by cause in NO_2_ hospitalization risk vary little by age group possibly because the two age groups, non-senior (1 to 65 years old, inclusive) versus seniors (≥ 66 years old), were too broad to capture age effect on NO_2_-related hospitalization risk.

Compared to age, sex differences in the adverse health effects of air pollution exposure have been examined less. Previous studies have reported sex-differences in mortality and hospitalization in response to short-term exposure to ambient air pollution with inconsistent results. In a study of 10 Italian cities, Chiusolo et al. [10], observed no effect modification by sex was observed for all-cause, cardiac, cerebrovascular, and respiratory mortality. A Brazilian study found sex-differences were not significant; however, associations with all-cause and respiratory mortality were stronger among women, while the opposite was true for cardiovascular mortality [30]. In contrast, a study of 272 Chinese cities [6] reported higher associations for females between NO_2_ and all-cause and cardiovascular mortality. Similarly, a Canadian study [16] found pollutant-season-specific sex differences in circulatory and respiratory hospitalization. They reported that the effect of sex was mixed across 24 Canadian cities where females had higher risk of respiratory hospitalization from NO_2_ exposures. In the current study population, males accounted for a larger proportion of circulatory (57% vs. 43%) and respiratory (52% vs. 48%) hospitalizations (Table 1). In particular, males accounted for a higher proportion of IHD hospitalizations in the Western region (67% vs. 64–65%) (Table 2). Even though age and body weight are contributors, sex is the strongest independent factor impacting cardio-respiratory function, as described by Brooks et al. [31]. Altogether, the biological sex differences in circulatory and respiratory structures and functions may underlie the observed regional differences in the Western region.

This study reported more visible regional differences in risk for circulatory hospitalizations and during the warm season. The Eastern region seems to have driven the regional differences, as it was usually at the margin of the national reference range. This indicates that Eastern Canada had more temporal variations, and deviated from the national risks of NO_2_-related acute hospitalizations during the study period. These variations can be explained by the relatively smaller population size (24% vs. 30–48% in Table S1) and lower hospitalizations rates (6,622 vs. 6,687–7,236 in Table 1). Estimates could be improved by including more urban or rural areas in Eastern Canada in future studies. However, the observed increasing deviations from the national risk estimates indicates under- or over-estimates which should be considered when extending national risks to the Eastern region.

The results reported in this article demonstrate that NO_2_-related acute hospitalizations were stable over time and that overall the regional estimates were within the national risk ranges. Nonetheless, there are many factors which can influence not only NO_2_ but also other air pollutant related hospitalization risk over time. Considering the potential changes (positive or negative) in these external factors, it is more suitable to investigate temporal trends in NO_2_-related acute hospitalization through dynamic models rather than accept the static models’ assumption of no trend. For the trend in health effects of NO_2_ over 17 years, socioeconomic changes (e.g., demographics, disease diagnosis, and medical and healthcare systems) and environmental background changes (e.g., climate change, freshwater shortages, biodiversity change) may also contribute to the change in hospitalizations. These factors are not daily variables and thus not taken into account for in this study.

Several limitations regarding design, exposure assessment, and consideration of gender exist in our approach. First, this ecological time-series study design was based on daily data aggregated across 24 cities implying that the unit of our analysis was city, not individual; that only day-to-day varying variables were included in the model for short-term exposure; and that primarily urban areas were examined. The study findings therefore may not represent the exposure-hospitalization relationship at the individual level, for long-term exposure, or for rural areas.

A second limitation is the classification of the 24 cities into three regions. While the Central region covers only the province of Ontario, the Western and Eastern regions each include several provinces. This is solely due to the fact that Ontario only covers about 37% of the Canadian population. Our regional classification was mainly based on geographical location, considering the population coverage among the regions: Central (48%), Western (30%), and Eastern (24%) of the study population. Spanning multiple provinces, cities in the Western and Eastern regions are more diverse socioeconomically and environmentally (industry distribution, sources of air pollution, climate, etc.). Zeng et al. [32] pointed out that not only geographical location but also geographic features (mountains and rivers) influenced NO_2_, which was not considered in our region classification. The study design may not capture the regional diversity in this large regional context, given the variability we know exists. We need to consider the heterogeneity within the regions when interpreting regional differences in this study.

A third limitation is the exposure assessment, since we assigned city average concentrations to all residents. This is known to introduce exposure misclassification and has been described by Klepeis et al. [33], Brown et al. [34], and Zeger et al. [35]. The degree of exposure misclassification depends on the spatial variability of NO_2_ within each city and is potentially high for a local, traffic-related air pollutant such as NO_2_. However, if individuals move about widely within their city, then error would be reduced. Alternatively, residential neighborhood (e.g., postal code) exposures could be assigned, if available, but this approach disregards the influence of individual mobility within the larger community. Alternative data sources such as land use regression models and satellite observations can also provide better spatial resolution (e.g., in 10 by 10 km), but lack the temporal resolution required for time series studies and depend heavily on models [36, 37]. Examining personal exposures are interesting but impractical in terms of time and cost. Other sources of exposure misclassification include behavioral factors such as air conditioner use in the warm season, reported on by Bell et al. [38] and Medina- Ramón & Schwartz [39] and time spent outdoors in the cold season, which were not controlled for in this study.

A fourth limitation is not accounting for co-pollutants in the model. Previous Canadian studies using multi-pollutant models (NO_2_, ground-level ozone, PM_2.5_ for 2001–2012) reported little additive or antagonistic risk of circulatory [36] and respiratory [38] health outcomes and explained it in relation to weak-to-moderate correlations among the three air pollutants. Over-dispersion Poisson regression models with 1-, 2-, or 3-pollutants did not change the significance and strength of the effect of one air pollutant after accounting for others, indicating that the 1-pollutant model did not result in considerable under- or over-estimates. We applied single-pollutant models for NO_2_ in this study based on these findings, however, missing co-pollutants remain as a limitation for two reasons. First, previous studies did not examine more specific causes under circulatory and respiratory health outcomes. For example, what was found for the broader category of circulatory hospitalizations may not be applied to the more specific causes (IHD, OHD or CEV) analyzed in this study. Second, interactions among co-pollutants cannot be explained by their correlations solely.

Finally, it is quite difficult to distinguish between sex and gender in epidemiologic studies [16]. Sex affects many physiological functions and disorders, including circulatory and respiratory diseases. On the other hand, gendered lifestyle factors such as occupation and socioeconomic status, and behaviours such as smoking, diet, and physical activity, may also be responsible for the observed differences in risk from air pollution exposures between men and women, reported by Matz et al. [40], Redline & Gold [41], London et al. [42], and Vlassoff [43]. We considered only sex, mainly due to the inability to distinguish sex from gender with the available data. The study findings on sex difference could be related to gender.

Notwithstanding the limitations, this study has many strengths. It provides a comprehensive analysis of regional differences in public health outcomes with temporal trends which could be used to improve the communication of adverse health effects of NO_2_ by adding region-specific information. Others have previously examined spatial and temporal patterns in mortality related to NO_2_ at the national level [1], but to our knowledge this is the first study that has examined more specific sub-causes (IHD, OHD, CEV, CLRD and InfPn) and regional differences in NO_2_-related hospitalization risks by age group and sex. Also, the study findings are based on a diverse sample across Canada which supports the validity of its findings. We used historical data for 17 years: hourly NO_2_ data (148,920 h) and daily hospitalization (6,205 days) for each city, thereby strengthening statistical power and stability, and temporal trends in the hospitalization risk estimates. Finally, we used NO_2_ concentrations measured by multiple ground-monitoring stations (not predicted by models) located in populated areas within each city. This exposure assignment can avoid issues such as heavy model-dependency and misclassification of NO_2_ exposures, which may result in unidentifiable bias in association estimates.

## Conclusion

This study identified regional differences in the adverse health effects of NO_2_ by cause, age, sex, and trend. We examined cause-specific hospitalizations such as IHD, OHD, CEV (circulatory system), and CLRD and InfPn (respiratory system). Further, the study presents trends and changes in regional differences, and in demographics (population, sex), health outcomes (circulatory- and respiratory-related hospitalizations), and environmental factors (NO_2_ and temperature).

Future studies including more cities, using more community-related information such as socioeconomic status, and identifying factors underlying these regional differences such as access to health care and climate change are needed to determine the causal mechanisms for these regional differences.

## Supplementary Information

Supplementary Material 1. Additional tables and figures describing the study population and estimated trends in hospitalization risk by region

## Data Availability

The air pollution and temperature datasets analyzed during the current study are open to the public by Environment and Climate Change Canada [http://data.ec.gc.ca/data/air/monitor/national-air-pollution-surveillance-naps-program/ and https://climatedata.ca/]. The health data used for this study cannot be shared freely due to the policy of the Canadian Institute for Health Information (CIHI) for the use and distribution of sensitive records. Our data source, the Discharge Abstract Database (DAD), captures administrative, clinical and demographic information on hospital discharges. Details regarding the DAD can be found at: https://www.cihi.ca/en/discharge-abstract-database-metadata-dad. The CIHI is the primary custodian of the DAD. CIHI is an independent, not-for-profit organization that provides essential information on Canada’s health systems and the health of people living in Canada. More information about CIHI can be found at: https://www.cihi.ca/en/about-cihi. Restrictions apply to the availability of the data as the data was used under a data sharing agreement and is not publicly available. General data inquiries about accessing CIHI data can be made at privacy@cihi.ca at the CIHI (https://www.cihi.ca/en/access-data-and-reports/data-holdings/make-a-data-request) or the corresponding author (hwashin.shin@hc-sc.gc.ca).

## Abbreviations

CD: Census division
CP: Cardiopulmonary
ICD: International Classification of Diseases
IHD: Ischemic heart disease
OHD: Other heart disease
CEV: Cerebrovascular disease
InfPn: Influenza/Pneumonia
CLRD: Chronic lower respiratory disease
NAPS: National Air Pollution Surveillance
CESI: Canadian Environmental Sustainability Indicators
