# Supplementary material for: Regional differences in acute hospitalization risk associated with NO2 by cause, season, age, sex, and trend: an ecological time series study in Canada
Source: BMC Public Health. 2025 Mar 31;25:1217. doi: 10.1186/s12889-025-22339-6 (PMC11956404; doi:10.1186/s12889-025-22339-6)
Supplement: Supplementary file 1 — Supplementary Material 1. Additional tables and figures describing the study population and estimated trends in hospitalization risk by region [file 12889_2025_22339_MOESM1_ESM.pdf]

Supplementary Materials

Regional Differences in Acute Hospitalization Risk Associated with NO_2 by Cause, Season, Age, Sex, and Trend: an Ecological Time Series Study in Canada.

Hwashin H. Shin[a,b,*], James Owen[a], Kimberly Mitchell[a], Marc Smith-Doiron[a], Parvin Dehghani[a]

[a] Environmental Health Science and Research Bureau, Health Canada, Ottawa, ON, Canada
[b] Department of Mathematics and Statistics, Queen's University, Kingston, ON, Canada

*Corresponding author: Hwashin.Shin@ hc-sc.gc.ca

Table of Contents

Table S1. Study population ratio by region for 1996-2012 2
Table S2. Relative changes in annual population by age and sex for 1996-2012 3
Table S3W. Annual ratio of hospitalization counts to population by sex and season for 1996-2012 (Western Canada) 4
Table S3C. Annual ratio of hospitalization counts to population by sex and season for 1996-2012 (Central Canada) 5
Table S3E. Annual ratio of hospitalization counts to population by sex and season for 1996-2012 (Eastern Canada)....................................6
Table S4W. Annual ratio of hospitalization counts to population by cause, sex and season for 1996-2012 (Western Canada)....................................7
Table S4C. Annual ratio of hospitalization counts to population by cause, sex and season for 1996-2012 (Central Canada)....................................8
Table S4E. Annual ratio of hospitalization counts to population by cause, sex and season for 1996-2012 (Eastern Canada)....................................9

Figure S1. Trends in estimated associations between NO_2 and hospitalizations related to IHD, OHD, and CEV during the warm and cold seasons....................................10
Figure S2. Trends in estimated associations between NO_2 and hospitalizations related to InfPn, and CLRD during the warm and cold seasons....................................11
Figure S3. Trends in regional estimates for cold season risk of circulatory hospitalization associated with a 10 ppb increase in NO_2, by age and sex....................................12
Figure S4. Trends in regional estimates for warm season risk of respiratory hospitalization associated with a 10 ppb increase in NO_2, by age and sex....................................13

Note: The order of materials in the Supplementary Materials follows the order of the main text.

Table S1. Study population and ratios to the national population, and proportions belonging to each region, from 1996-2012.

Year	Study Population		Regional Proportion		
	N[1]	Ratio to National Population[2] (%)	Western Canada[3] (%)	Central Canada[3] (%)	Eastern Canada[3] (%)
1996	14,762,948	50	29	47	25
1997	14,975,661	50	30	47	25
1998	15,168,919	50	30	47	25
1999	15,366,134	50	30	47	24
2000	15,599,481	51	30	47	24
2001	15,871,729	51	29	48	24
2002	16,127,769	51	29	48	24
2003	16,311,527	51	29	48	24
2004	16,496,600	51	29	48	24
2005	16,687,536	52	30	48	24
2006	16,892,477	52	30	48	23
2007	17,079,834	52	30	48	23
2008	17,295,371	52	30	48	23
2009	17,540,449	52	30	48	23
2010	17,777,973	52	30	48	23
2011	17,997,517	52	30	48	23
2012	18,287,658	52	31	48	23
**Combined[4] (SD[5])**	**16,484,681 (1,092,194)**	**52 (1.2)**	**30 (0.4)**	**48 (0.4)**	**24 (0.7)**
**CV[6]**	**0.07**	**0.02**	**0.01**	**0.01**	**0.03**

[1] Total population of the 24 selected cities combined
[2] (Study population/National population)*100
[3] (Study regional population/Study population)*100. The sum of 3 regions is close to 100% due to rounding errors.
[4] Average over 17 years
[5] Standard deviation over 17 years
[6] Coefficient of variation=(SD/average), a statistical measure of the dispersion of data around the mean.

Table S2. Relative changes in annual population by age and sex for 1996-2012

		All ages ≥ 1			Seniors ≥ 66		
**Region**	**City**	**Annual population in 1000's (percent change[a])**	**%[b] female (Trend[c])**	**%[b] male (Trend[c])**	**%[b] seniors (Trend[a])**	**%[b] female (Trend[c])**	**%[b] male (Trend[c])**
**Eastern Canada**		**3,698 - 4,181 (13)**	**51 - 51 (0)**	**49 - 49 (0)**	**477 - 624 (31)**	**61 - 59 (-)**	**39 - 41 (+)**
	Halifax	351 - 407 (16)	51 - 51 (0)	49 - 49 (0)	36 - 54 (50)	59 - 56 (-)	41 - 44 (+)
	Saint John	74 -70 (-5)	52 - 52 (0)	48 - 48 (0)	11 - 12 (9)	62 - 59 (-)	38 - 41 (+)
	Quebec	513 - 567 (11)	51 - 51 (0)	49 - 49 (0)	65 - 103 (58)	63 - 58 (-)	37 - 42 (+)
	Montreal	1,798 – 1,941 (8)	52 - 50 (-)	48 - 50 (+)	266 - 299 (12)	61 - 59 (-)	39 - 41 (+)
	Ottawa	962 – 1,196 (24)	51 - 51(0)	49 - 49 (0)	99 - 156 (58)	60 - 57 (-)	40 - 43 (+)
**Central Canada**		**6,947 - 8,786 (26)**	**51 - 51 (0)**	**49 - 49 (0)**	**806 - 1,201 (49)**	**58 - 56 (-)**	**42 - 44 (+)**
	Durham	472 - 636 (35)	50 - 50 (0)	50 - 50 (0)	42 - 79 (88)	57 - 55 (-)	43 - 45 (+)
	York	611 – 1,086 (**78**)	50 - 50 (0)	50 - 50 (0)	50 - 131 (**162**)	56 - 54 (-)	44 - 46 (+)
	Toronto	2,456 – 2,741 (12)	51 - 51 (0)	49 - 49 (0)	324 - 390 (20)	58 - 57 (-)	42 - 43 (+)
	Peel	879 – 1,365 (55)	49 - 49 (0)	51 - 51 (0)	63 - 146 (**132**)	57 - 54 (-)	43 - 46 (+)
	Halton	350 - 528 (51)	50 - 51 (+)	50 - 49 (-)	38 - 71 (87)	56 - 56 (0)	44 - 44 (0)
	Hamilton	481 - 541 (12)	50 - 50 (0)	50 - 50 (0)	67 - 85 (27)	58 - 56 (-)	42 - 44 (+)
	Niagara	415 - 444 (7)	51 - 51 (0)	49 - 49 (0)	65 - 85 (31)	57 - 55 (-)	43 - 45 (+)
	Waterloo	417 - 530 (27)	50 - 50 (0)	50 - 50 (0)	45 - 67 (49)	59 - 56 (-)	41 - 44 (+)
	Windsor	204 - 218 (7)	51 - 51 (0)	49 - 49 (0)	29 - 35 (21)	60 - 56 (-)	40 - 44 (+)
	Sarnia	75 - 74 (-1)	51 - 51 (0)	49 - 49 (0)	11 - 14 (27)	58 - 57 (-)	42 - 43 (+)
	London	335 - 382 (14)	51 - 51 (0)	49 - 49 (0)	41 - 56 (37)	59 - 57 (-)	41 - 43 (+)
	Sudbury	170 - 165 (-3)	50 - 50 (0)	50 - 50 (0)	20 - 27 (35)	56 - 56 (0)	44 - 44 (0)
	Sault Ste Marie	82 - 76 (-7)	51 - 51 (0)	49 - 49 (0)	11 - 15 (36)	57 - 56 (-)	43 - 44 (+)
**Western Canada**		**4,340 - 5,591 (29)**	**50 - 50 (0)**	**50 - 50 (0)**	**489 - 696 (42)**	**58 - 56 (-)**	**42 - 44 (+)**
	Winnipeg	629 - 690 (10)	51 - 50 (-)	49 - 50 (+)	85 - 98 (15)	60 - 58 (-)	40 - 42 (+)
	Regina	186 - 206 (11)	51 - 50 (-)	49 - 50 (+)	22 - 27 (23)	60 - 58 (-)	40 - 42 (+)
	Saskatoon	199 - 239 (20)	51 - 50 (-)	49 - 50 (+)	23 - 29 (26)	60 - 58 (-)	40 - 42 (+)
	Calgary	789 – 1,179 (49)	49 - 49 (0)	51 - 51 (0)	69 - 116 (68)	57 - 56 (-)	43 - 44 (+)
	Edmonton	632 - 869 (38)	50 - 49 (-)	50 - 51 (+)	69 - 99 (43)	58 - 56 (-)	42 - 44 (+)
	Vancouver	1,905 – 2,408 (26)	50 - 50 (0)	50 - 50 (0)	221 - 327 (48)	57 - 54 (-)	43 - 46 (+)
**Combined**		**14,985 - 18,558 (24)**	**51 - 51 (0)**	**49 - 49 (0)**	**1,772 - 2,521 (42)**	**58 - 56 (-)**	**42 - 44 (+)**

[a] percent change: [(count in 2012 – count in 1996) / (count in 1996)] * 100

[b] % (i.e. count/population*100) in 1996-2012

[c] Trend direction: + for increased ; – for decreased; and 0 for no change

Table S3W. Annual hospitalization counts and rates, and proportions by sex and season for 1996-2012 **(Western Canada)**

	**All non-accidental (ICD10, A00-R99)**				**Circulatory (ICD-10, I00-I99)**				**Respiratory (ICD10, J00-J99)**			
Year	Count	rate per 100k[1]	% female[2]	% in warm[3]	rate per 100k[1]	% of all causes[4]	% female[2]	% in warm[3]	rate per 100k[1]	% of all causes[4]	% female[2]	% in warm[3]
1996	367,175	8,460	61	50	1,251	15	44	50	836	10	47	44
1997	363,063	8,207	62	50	1,214	15	44	50	791	10	47	44
1998	361,272	8,041	62	49	1,188	15	43	49	811	10	48	41
1999	358,597	7,871	61	49	1,163	15	43	49	850	11	48	40
2000	350,748	7,597	62	49	1,144	15	43	49	706	9	47	44
2001	336,693	7,191	62	49	1,075	15	43	48	655	9	47	45
2002	332,970	7,010	62	49	1,024	15	43	49	633	9	47	44
2003	339,388	7,066	62	50	997	14	42	49	647	9	47	44
2004	340,973	7,016	61	49	960	14	42	49	629	9	48	44
2005	349,217	7,076	61	50	941	13	42	50	672	10	48	46
2006	349,107	6,953	61	50	894	13	42	49	618	9	47	45
2007	350,299	6,866	61	50	852	12	42	49	592	9	47	45
2008	353,382	6,805	62	50	847	12	42	49	587	9	47	45
2009	360,772	6,804	62	50	831	12	42	49	593	9	47	44
2010	366,405	6,798	61	50	838	12	42	49	572	8	47	46
2011	377,410	6,902	61	50	839	12	42	50	592	9	47	45
2012	386,657	6,915	61	50	833	12	42	50	601	9	48	45
Western Canada (SD[5])	355,537	7,236 (545)	61 (0.3)	50 (0.4)	983 (152)	14 (1.2)	42 (0.7)	49 (0.4)	664 (94)	9 (0.6)	47 (0.3)	44 (1.5)

[1] hospitalization rate: (Regional hospitalization counts/ Regional population) *100,000

[2] (hospitalization counts for females (≥1 year old) / hospitalization counts for both sexes (≥1 year old)) *100. This includes admissions for childbirth.

[3] (hospitalization counts in the warm season (April-September) / year-round (January-December) counts) *100

[4] (specific-cause hospitalizations/study all-cause counts)*100, where study all-cause counts are in the 2nd column.

[5] Standard Deviation over 17 years.

Table S3C. Annual hospitalization counts and rates, and proportions by sex and season from 1996-2012 **(Central Canada)**

	All non-accidental (ICD10, A00-R99)				Circulatory (ICD-10, I00-I99)				Respiratory (ICD10, J00-J99)			
Year	Count	rate per 100k[1]	% female[2]	% in warm[3]	rate per 100k[1]	% of all causes[4]	% female[2]	% in warm[3]	rate per 100k[1]	% of all causes[4]	% female[2]	% in warm[3]
1996	583,401	8,399	61	49	1,407	17	44	50	774	9	47	42
1997	558,140	7,902	61	50	1,376	17	43	50	692	9	47	43
1998	553,839	7,721	61	50	1,343	17	44	49	709	9	47	40
1999	550,793	7,569	60	50	1,298	17	44	50	732	10	47	39
2000	537,252	7,251	60	50	1,267	17	44	49	647	9	47	42
2001	545,581	7,206	61	50	1,234	17	43	50	624	9	47	44
2002	534,900	6,926	60	50	1,164	17	42	49	592	9	47	44
2003	512,788	6,547	60	48	1,067	16	42	48	564	9	47	41
2004	528,298	6,648	60	50	1,066	16	42	49	546	8	47	44
2005	528,558	6,562	60	50	1,015	15	42	49	583	9	47	43
2006	504,030	6,171	60	49	970	16	42	49	534	9	47	44
2007	496,917	6,016	60	50	935	16	41	49	509	8	47	44
2008	494,919	5,921	60	50	931	16	42	49	510	9	47	45
2009	498,883	5,900	60	50	917	16	42	50	525	9	47	43
2010	503,050	5,874	60	50	921	16	42	49	510	9	47	44
2011	514,441	5,935	59	50	905	15	42	49	539	9	47	44
2012	531,593	6,050	59	49	921	15	41	49	548	9	48	42
Central Canada (SD[5])	528,081	6,687 (808)	60 (0.5)	50 (0.4)	1,090 (183)	16 (0.8)	42 (0.9)	49 (0.4)	591 (85)	9 (0.3)	47 (0.3)	43 (1.6)

[1] hospitalization rate: (Regional hospitalization counts/ Regional population) *100,000

[2] (hospitalization counts for females (≥1 year old) / hospitalization counts for both sexes (≥1 year old)) *100. This includes admissions for childbirth.

[3] (hospitalization counts in the warm season (April-September) / year-round (January-December) counts) *100

[4] (specific-cause hospitalizations/study all-cause counts)*100, where study all-cause counts are in the 2nd column.

[5] Standard Deviation over 17 years.

Table S3E. Annual hospitalization counts and rates, and proportions by sex and season from 1996-2012 **(Eastern Canada)**

	**All non-accidental (ICD10, A00-R99)**				**Circulatory (ICD-10, I00-I99)**				**Respiratory (ICD10, J00-J99)**			
Year	Count	rate per 100k[1]	% female[2]	% in warm[3]	rate per 100k[1]	% of all causes[4]	% female[2]	% in warm[3]	rate per 100k[1]	% of all causes[4]	% female[2]	% in warm[3]
1996	280,178	8,058	61	50	1,347	17	45	49	745	9	50	43
1997	269,017	7,711	60	50	1,338	17	45	50	749	10	49	42
1998	264,128	7,540	60	50	1,299	17	45	49	752	10	50	40
1999	256,080	7,247	60	48	1,250	17	45	48	761	10	50	37
2000	248,173	6,945	60	49	1,258	18	45	49	667	10	50	40
2001	240,003	6,633	61	49	1,176	18	45	48	623	9	50	42
2002	229,290	6,274	61	50	1,120	18	45	49	579	9	49	43
2003	229,751	6,250	60	50	1,098	18	43	49	567	9	49	45
2004	233,298	6,323	60	49	1,088	17	43	49	603	10	50	42
2005	234,781	6,349	59	50	1,063	17	42	49	628	10	49	42
2006	232,442	6,275	60	49	1,028	16	43	48	580	9	49	44
2007	228,568	6,148	60	49	997	16	42	49	543	9	50	44
2008	232,972	6,222	60	50	963	15	42	49	578	9	50	45
2009	233,298	6,167	60	50	928	15	44	49	573	9	50	43
2010	237,540	6,212	60	50	913	15	43	50	563	9	50	45
2011	241,224	6,247	60	50	901	14	43	49	585	9	50	43
2012	245,229	6,274	60	49	893	14	44	49	604	10	50	42
Eastern Canada (SD[5])	243,292	6,622 (620)	60 (0.3)	50 (0.5)	1,093 (157)	16 (1.3)	44 (1.0)	49 (0.5)	627 (76)	9 (0.4)	50 (0.4)	42 (2.0)

[1] hospitalization rate: (Regional hospitalization counts/ Regional population) *100,000

[2] (hospitalization counts for females (≥1 year old) / hospitalization counts for both sexes (≥1 year old)) *100. This includes admissions for childbirth.

[3] (hospitalization counts in the warm season (April-September) / year-round (January-December) counts) *100

[4] (specific-cause hospitalizations/study all-cause counts)*100, where study all-cause counts are in the 2nd column.

[5] Standard Deviation over 17 years.

Table S4W. Annual rates of hospitalization by cause, and proportions by sex and season, from 1996-2012 **(Western Canada)**

	**Ischemic Heart Disease (IHD, ICD-10, I20-I25)**			**Other forms of Heart Diseases (OHD, ICD-10, I30-I52)**			**Cerebrovascular Diseases (CEV, ICD-10, I60-I69)**			**Chronic Lower Respiratory Diseases (CLRD, ICD-10, J40-J47)**			**Influenza and Pneumonia (InfPn, ICD-10, J09-J18)**		
Year	rate per 100k[1]	% female[2]	% in warm[3]	rate per 100k[1]	% female[2]	% in warm[3]	rate per 100k[1]	% female[2]	% in warm[3]	rate per 100k[1]	% female[2]	% in warm[3]	rate per 100k[1]	% female[2]	% in warm[3]
1996	456	35	50	367	49	49	194	50	50	261	49	47	252	47	42
1997	438	34	50	366	49	49	188	51	50	253	50	49	248	47	41
1998	431	34	49	364	48	49	187	51	50	257	50	43	288	48	38
1999	427	34	50	355	47	48	186	50	49	282	50	43	300	48	36
2000	428	33	49	341	48	49	185	51	51	223	50	48	239	47	41
2001	414	34	49	319	49	48	165	52	48	212	50	48	222	48	45
2002	409	34	49	309	49	50	145	50	48	210	50	48	205	48	41
2003	394	33	49	300	48	49	140	50	50	222	50	46	201	48	42
2004	377	33	49	291	48	50	138	49	50	241	49	46	173	50	42
2005	355	32	50	300	48	50	140	49	49	267	49	49	182	50	44
2006	332	32	49	291	48	48	136	49	49	241	49	47	163	49	42
2007	312	32	49	281	47	49	129	50	50	232	50	46	157	48	44
2008	297	32	49	289	46	49	128	49	50	227	49	46	159	49	42
2009	279	31	49	293	47	49	126	48	49	213	50	47	181	49	42
2010	276	31	49	301	48	49	128	48	51	218	50	49	150	50	44
2011	269	30	50	306	48	50	130	46	49	221	51	46	157	49	43
2012	263	31	50	312	47	50	130	47	50	226	51	46	160	49	44
**Western**	357	33	49	315	48	49	150	49	50	235	50	47	199	48	42

[1] hospitalization rate: (Study all hospitalization counts/ Study population) *100,000

[2] (hospitalization counts for females (≥1 year old) / hospitalization counts for both sexes (≥1 year old))*100

[3] (hospitalization counts in the warm season (April-September) / year-round (January-December) counts) *100

Table S4C. Annual rates of hospitalization by cause, and proportions by sex and season, from 1996-2012 **(Central Canada)**

	**Ischemic Heart Disease (IHD, ICD-10, I20-I25)**			**Other forms of Heart Diseases (OHD, ICD-10, I30-I52)**			**Cerebrovascular Diseases (CEV, ICD-10, I60-I69)**			**Chronic Lower Respiratory Diseases (CLRD, ICD-10, J40-J47)**			**Influenza and Pneumonia (InfPn, ICD-10, J09-J18)**		
Year	rate per 100k[1]	% female[2]	% in warm[3]	rate per 100k[1]	% female[2]	% in warm[3]	rate per 100k[1]	% female[2]	% in warm[3]	rate per 100k[1]	% female[2]	% in warm[3]	rate per 100k[1]	% female[2]	% in warm[3]
1996	570	37	50	384	48	49	210	50	50	247	50	44	221	48	40
1997	579	37	50	382	48	50	200	49	50	222	49	47	195	47	41
1998	571	37	49	379	49	49	199	50	50	235	50	41	220	48	39
1999	567	37	50	361	48	49	190	51	49	246	49	42	231	47	36
2000	555	37	49	357	49	50	187	51	50	210	50	45	214	48	39
2001	545	36	50	351	49	49	179	51	50	205	49	47	198	49	42
2002	527	35	50	344	48	49	152	50	47	196	49	48	178	48	42
2003	475	34	48	323	47	48	138	50	49	199	49	43	166	49	38
2004	464	34	49	333	48	49	136	49	50	207	48	46	148	48	41
2005	423	34	49	328	48	49	135	50	48	230	49	45	154	49	41
2006	390	34	50	321	48	49	134	49	50	207	49	47	140	48	41
2007	366	33	48	317	47	50	130	48	49	193	49	45	133	48	43
2008	364	33	48	316	47	49	132	48	50	195	49	46	139	49	45
2009	345	32	50	322	48	49	130	48	50	186	49	45	160	49	39
2010	344	32	50	324	48	49	130	48	50	183	49	47	142	50	41
2011	328	32	49	327	48	49	127	49	49	188	50	47	160	49	41
2012	334	32	50	339	46	49	127	48	50	189	50	44	159	49	40
**Central**	449	35	49	340	48	49	153	49	50	207	49	45	172	48	40

[1] hospitalization rate: (Study all hospitalization counts/ Study population) *100,000

[2] (hospitalization counts for females (≥1 year old) / hospitalization counts for both sexes (≥1 year old))*100

[3] (hospitalization counts in the warm season (April-September) / year-round (January-December) counts) *100

Table S4E. Annual rates of hospitalization by cause, and proportions by sex and season, from 1996-2012 **(Eastern Canada)**

Year	**Ischemic Heart Disease (IHD, ICD-10, I20-I25)**			**Other forms of Heart Diseases (OHD, ICD-10, I30-I52)**			**Cerebrovascular Diseases (CEV, ICD-10, I60-I69)**			**Chronic Lower Respiratory Diseases (CLRD, ICD-10, J40-J47)**			**Influenza and Pneumonia (InfPn, ICD-10, J09-J18)**		
	rate per 100k[1]	% female[2]	% in warm[3]	rate per 100k[1]	% female[2]	% in warm[3]	rate per 100k[1]	% female[2]	% in warm[3]	rate per 100k[1]	% female[2]	% in warm[3]	rate per 100k[1]	% female[2]	% in warm[3]
1996	537	37	49	346	50	48	205	51	49	300	53	43	189	50	41
1997	531	38	50	365	49	49	205	51	49	299	51	44	200	49	39
1998	524	37	49	366	50	49	191	50	50	298	53	41	218	49	38
1999	522	38	49	336	51	48	184	52	48	305	53	38	216	51	34
2000	540	37	49	332	50	49	174	52	48	261	53	42	190	51	38
2001	501	37	49	323	51	48	161	52	49	240	53	43	185	51	42
2002	489	37	48	312	51	48	148	52	50	224	52	44	173	49	42
2003	481	35	49	308	49	49	142	52	50	228	52	47	150	50	43
2004	462	35	49	319	49	49	139	52	50	242	53	44	168	50	40
2005	437	34	49	315	47	49	145	50	50	245	53	42	178	49	41
2006	417	35	49	327	48	48	124	52	47	222	54	47	155	50	43
2007	393	34	48	325	48	48	124	49	50	206	54	44	146	49	44
2008	357	34	48	328	47	48	122	48	49	217	54	47	160	52	44
2009	329	34	50	331	48	49	116	51	49	203	55	46	172	51	41
2010	320	34	49	328	48	49	118	50	52	206	55	47	160	52	44
2011	296	33	50	341	48	49	118	51	50	202	55	45	180	51	42
2012	294	34	49	344	48	49	117	51	50	219	55	44	181	51	41
**Eastern**	434	36	49	332	49	49	148	51	49	241	53	44	177	50	41

[1] hospitalization rate: (Study all hospitalization counts/ Study population) *100,000

[2] (hospitalization counts for females (≥1 year old) / hospitalization counts for both sexes (≥1 year old))*100

[3] (hospitalization counts in the warm season (April-September) / year-round (January-December) counts) *100

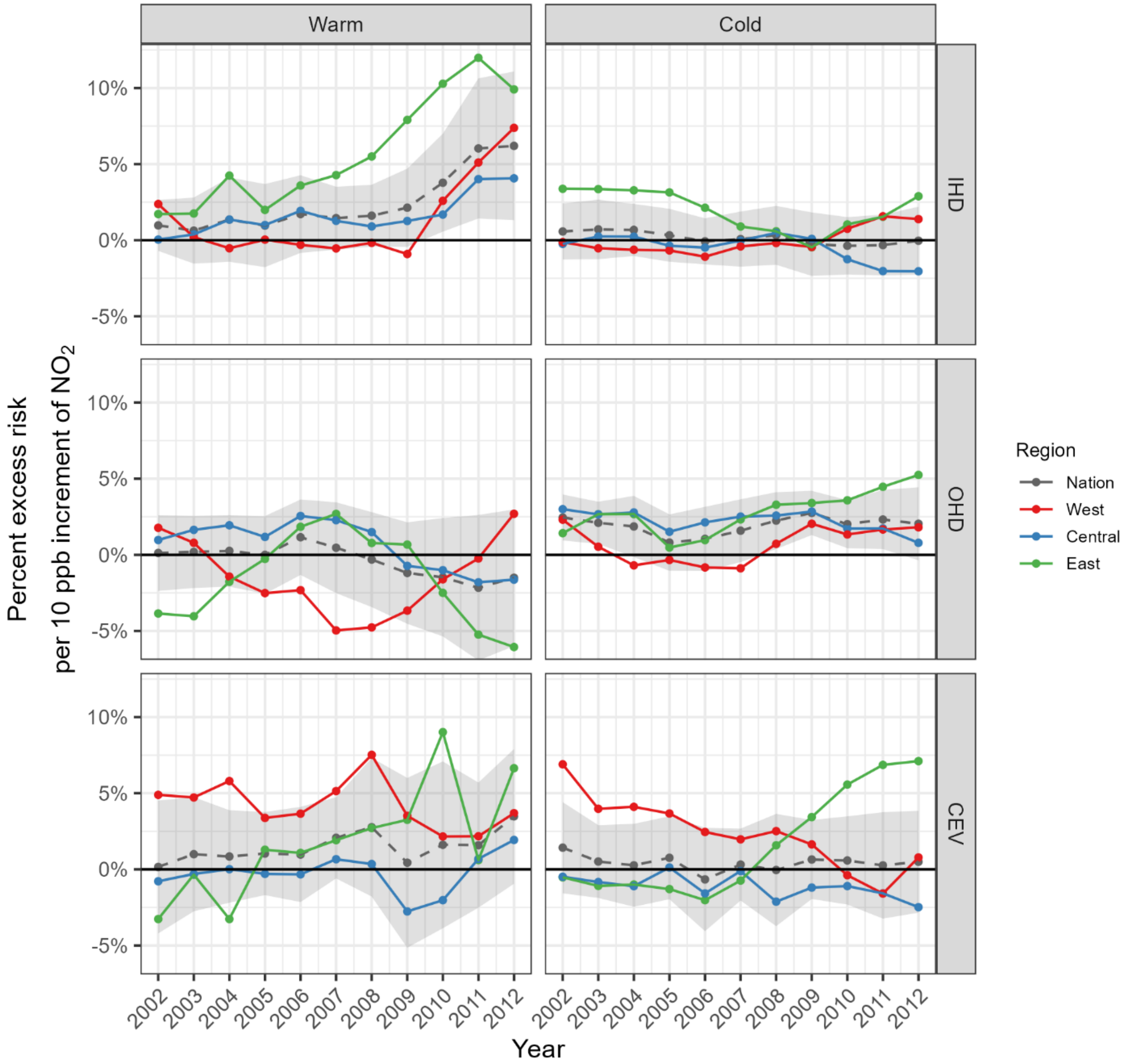

Figure S1. Trends in estimated associations between NO_2 and hospitalizations related to IHD, OHD, and CEV during the warm and cold seasons. A 95% credible interval for the estimated national risk is shown in grey. Ischemic heart disease (IHD, I20-I25); other heart disease (OHD, I30-I52); and cerebrovascular disease (CEV, I60-I69).

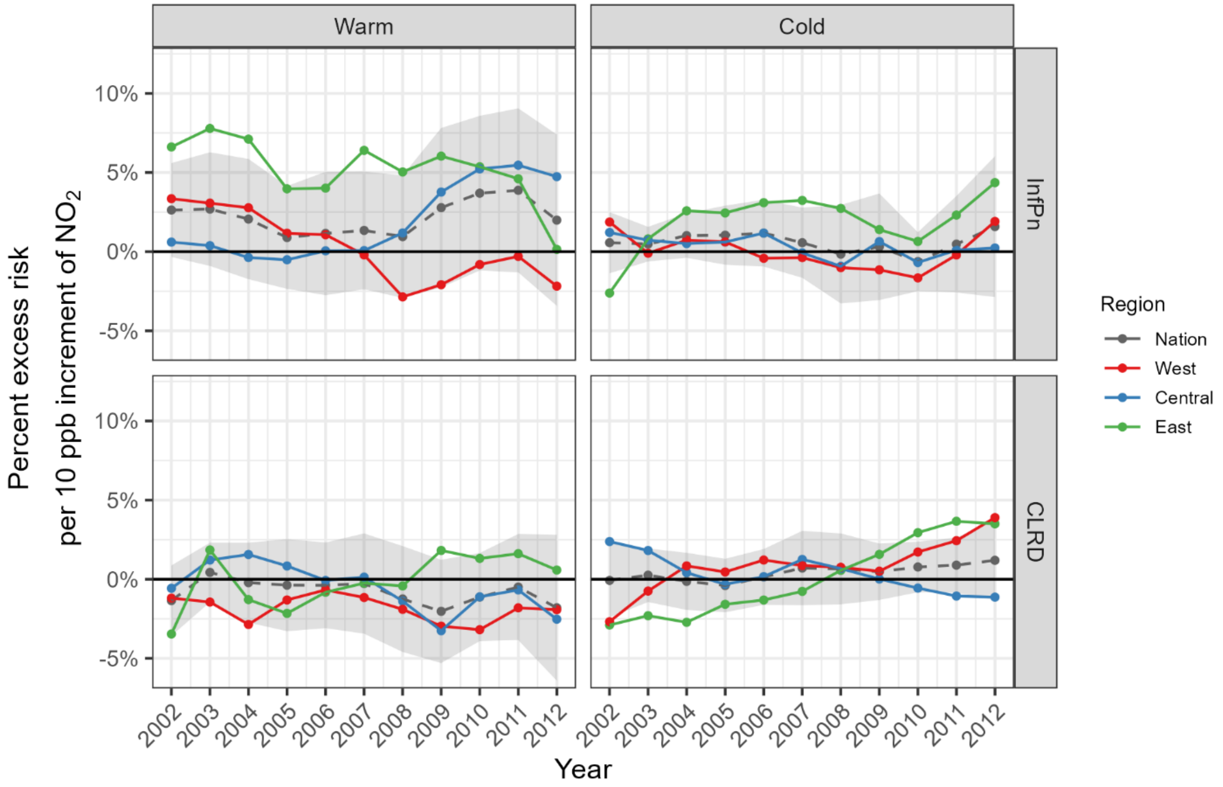

Figure S2. Trends in estimated associations between NO2 and hospitalizations related to InfPn, and CLRD during the warm and cold seasons. A 95% credible interval for the estimated national risk is shown in grey. Influenza/pneumonia (InfPn, J09-J18); and chronic lower respiratory diseases (CLRD, J40-J47).

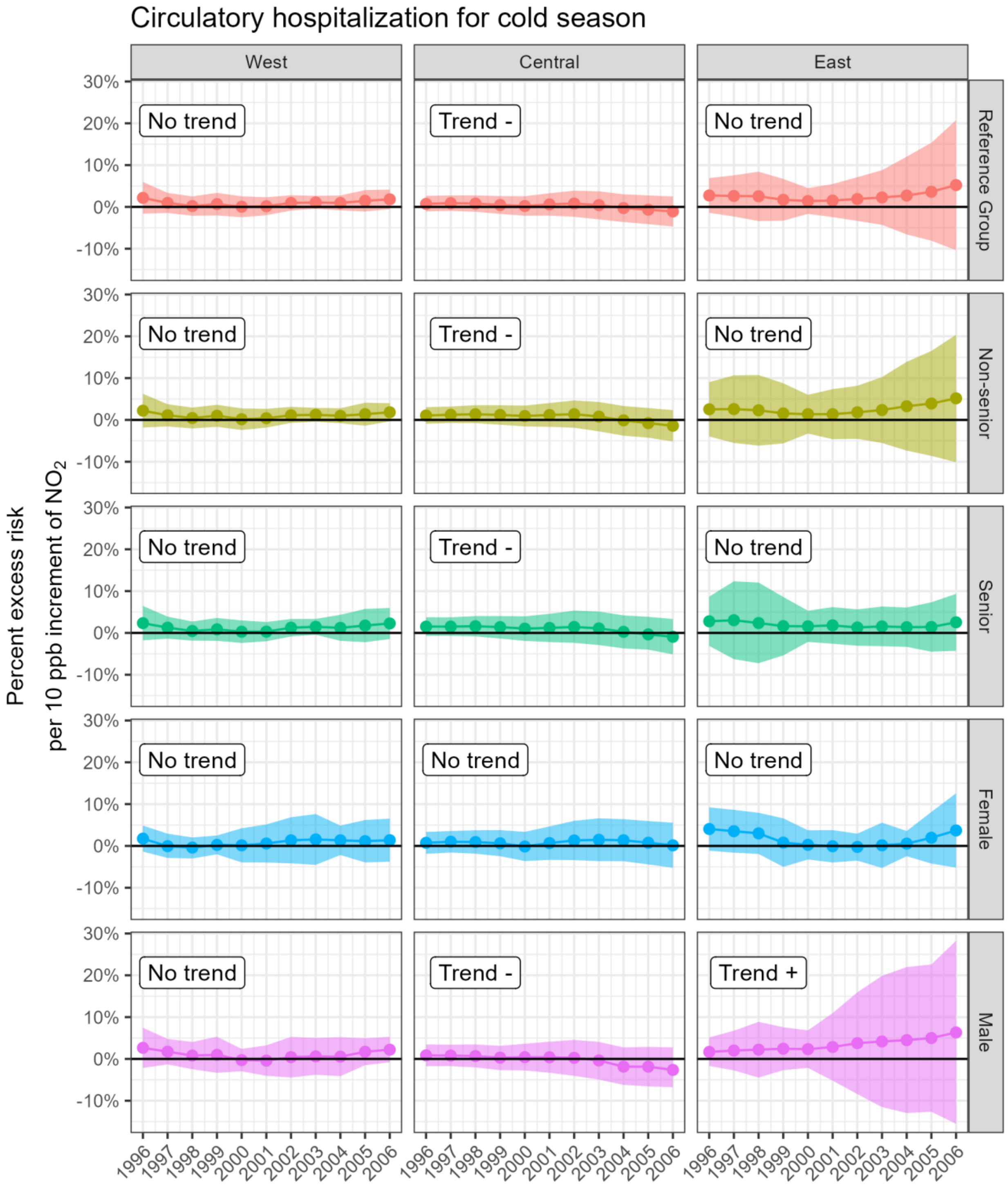

Figure S3. Trends in regional estimates (with 95% credible intervals) for cold season risk of circulatory hospitalization associated with a 10 ppb increase in NO_2, by age and sex. Trend is indicated based on Sen's slope (with 95% confidence level); No trend: no linear trend; Trend -: decreasing linear trend; and Trend +: increasing linear trend.

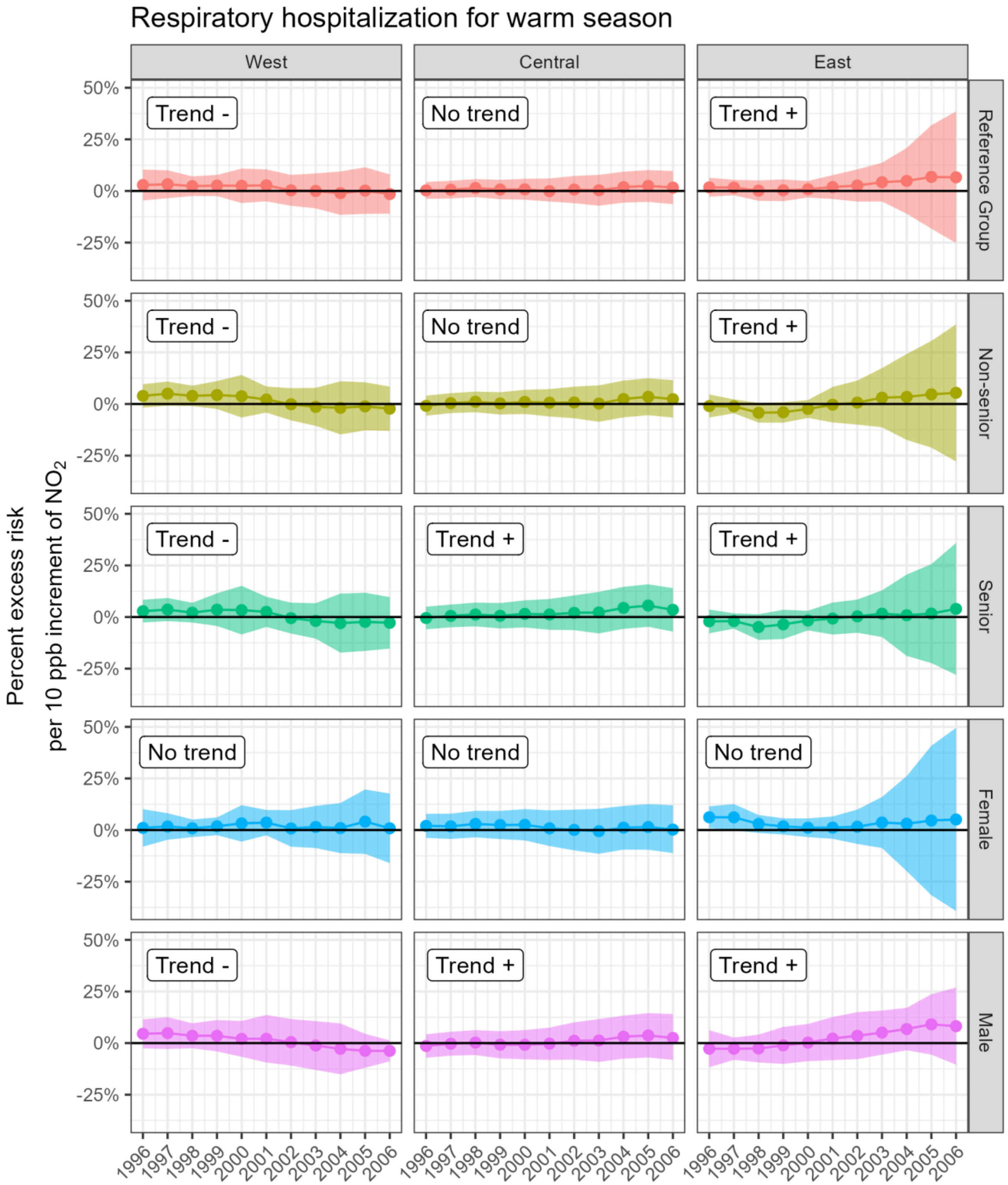

Figure S4. Trends in regional estimates (with 95% credible intervals) for warm season risk of respiratory hospitalization associated with a 10 ppb increase in NO_2, by age and sex. Trend is indicated based on Sen's slope (with 95% confidence level); No trend: no linear trend; Trend -: decreasing linear trend; and Trend +: increasing linear trend.
